# When *Cannabis sativa* L. Turns Purple: Biosynthesis and Accumulation of Anthocyanins

**DOI:** 10.3390/antiox12071393

**Published:** 2023-07-06

**Authors:** Laura Bassolino, Flavia Fulvio, Chiara Pastore, Federica Pasini, Tullia Gallina Toschi, Ilaria Filippetti, Roberta Paris

**Affiliations:** 1 CREA—Research Centre for Cereal and Industrial Crops, Via di Corticella 133, 40128 Bologna, Italy; flavia.fulvio@crea.gov.it; 2 Department of Agricultural and Food Sciences, University of Bologna, 40127 Bologna, Italy; chiara.pastore@unibo.it (C.P.); federica.pasini5@unibo.it (F.P.); tullia.gallinatoschi@unibo.it (T.G.T.); ilaria.filippetti@unibo.it (I.F.)

**Keywords:** *Cannabis sativa*, keracyanin, transcription factors, decorating enzymes, MYB, MATE, HPLC-MS/MS, circular economy

## Abstract

Environmental cues elicit anthocyanin synthesis in plant vegetative and reproductive tissues. Their accumulation in different organs accounts for their diverse biological functions, mainly related to their antioxidant properties, and it depends on a temporally and spatially regulated mechanism controlled by the action of a well-known multi-transcription factor complex. Despite the highly recognizable value of *Cannabis sativa* L. as a natural biorefinery of phytochemicals, very little information is known on anthocyanin pigmentation in this species. In this work, a targeted quantification of anthocyanins via HPLC-MS/MS, combined with the transcriptional profile via RT-qPCR of genes encoding for structural and decorating enzymes and regulatory transcription factors in different *C. sativa* tissues, help gain insights into the anthocyanin pathway in this species. To the best of our knowledge, this is the first report on the identification of cyanidin-3-rutinoside (keracyanin) as the major anthocyanin in *C. sativa* vegetative and floral tissues. Keracyanin amounts were higher than in small berries, suggesting that *Cannabis* biomass is a valuable source of colored antioxidants to be exploited in diverse applications. Furthermore, a gene putatively encoding for an anthocyanin DTX35 type transporter and *CsTTG1* were identified in silico and their transcriptional levels were assessed via RT-qPCR. The results allow us to provide the first model of anthocyanin regulation in *C. sativa,* opening a new research scenario in this species for both breeding purposes and phytochemical exploitation.

## 1. Introduction

Anthocyanins are the major flower pigments, broadly distributed in land plants [1]. They play an ecological role in plants’ capability to interact within a changing environment and anthocyanin pigmentation is considered a “honest signal” for pollinators and seed dispersal agents of dietary reward and antioxidant content [2,3]. Furthermore, these secondary metabolites exert a defensive role in plants’ response to both biotic and abiotic cues, e.g., solar irradiation and pathogen attack [4,5].

Besides their biological functions, there is a wide interest in both health-promoting properties and industrial applications of polyphenols, and in particular of anthocyanins [6], due to their capability to scavenge reactive oxygen species (ROS) [7]. Indeed, these compounds can be used as active ingredients for several products, from nutraceuticals to natural dyes and food additives. Until now, research to develop nutraceutical products beneficial to human diets has been mainly focused on food crops; however, bioactive molecules from alternative plant sources (e.g., industrial crops) and from by-products of industrial cultivation can be relevant in a circular economy scenario.

Anthocyanins are water soluble compounds, stably stored in the plant cell vacuoles and synthesized through a branch of the flavonoid grid that uses the amino acid phenylalanine as precursor of the diphenylpropane skeleton (C3-C6-C3) [8,9]. Despite the conserved core of the five most abundant aglycones, there is a huge chemical diversity in anthocyanin structure, with more than 600 structures identified to date. These differ in their side-chain decoration patterns, that are usually taxa specific and account for unique biologically properties. Anthocyanin synthesis is one of the best examples of secondary metabolism grids, largely investigated, with genes encoding for structural enzymes identified both in model plants and crop species. Taking advantage of the high conservation among species and of the availability of genome sequencing data, which allows the in silico identification of ortholog sequences, the regulatory machinery controlling the timely and tissue specific accumulation of anthocyanins, composed of a complex of the three types of transcription factors MYB-WD40-bHLH, has also been deciphered [10,11]. Indeed, anthocyanin synthesis can be seen as a “model metabolic pathway” to study temporal and spatial control of gene expression in plants. Despite the efforts of characterizing the enzymatic routes leading to anthocyanin synthesis in several plant systems, there are still few studies addressing the compartmentation and spatial distribution of these secondary metabolites in plant cells and their catabolism. A fine understanding of genes encoding for decorating enzymes is required to specifically address the synthesis of selected anthocyanins via engineering approaches or breeding strategies [6,12].

*Cannabis sativa* L. is a known biorefinery of bioactive compounds, even though it is still poorly explored as a source of anthocyanins. Despite the phenotypic evidence of anthocyanin pigmentation in several genotypes and commercial strains, a robust biochemical and molecular characterization of anthocyanin traits in *Cannabis* is still lacking. In a previous work, we identified, through a genome wide analysis on cs10 genome, a core set of candidate genes for both the steps leading to anthocyanin synthesis in *C. sativa* tissues and their regulatory players represented by myeloblastosis (MYB) and basic-helix-loop-helix (bHLH) transcription factors [13].

In this work, we exploit a selection of genotypes with anthocyanin pigmentation to provide the first biochemical evidence of anthocyanin compounds in *C. sativa* via targeted HPLC-MS. Cyanidin-3-rutinoside (keracyanin) was identified as the major pigment in purple leaf, stem, and flower tissues. A targeted RT-qPCR profiling of genes encoding for biosynthetic enzymes and transcription factors was also performed to highlight candidate genes involved in keracyanin synthesis in selected tissues. 

In addition to the previously identified candidates, in this work we provide the first evidence of a putative MATE type transporter, DETOXIFICATION 35 (DTX35), involved in the import of anthocyanins-3-O-rutinoside in *Cannabis* plant cell vacuoles. 

## 2. Materials and Methods

### 2.1. Plant Material, Growth Conditions and Sampling 

Six different *C. sativa* genotypes, namely Fibrante, S1750, S1652, S1759, V18 and PurpleF2, were cultivated outdoor in an experimental field located in Bologna, Northern Italy (latitude: 44.535160 longitude: 11.3565) in the 2021season. Sowing took place manually on May 4. Row distance was set as 0.5 m, no herbicides or pesticides were applied during the growing cycle. The cultivation was carried out under rainfed conditions and supported with a drip irrigation system when needed. 

Green and pigmented samples for both chemical and molecular analysis were collected from two out of the six genotypes (Fibrante and S1750), as detailed in Table 1. These samples were taken in three biological replicates (n = 3). In addition, to enlarge the dataset for biochemical characterization, other samples diversified for pigmentation were collected as single samples from the other genotypes cultivated in the same plot test, as reported in Table 1. All samples were collected in liquid nitrogen and stored at −80 °C until analyses. 

### 2.2. Chemicals and Reagents

HPLC-grade methanol, acetonitrile, water, perchloric and formic acids were purchased from Merck KGaA (Darmstadt, Germany). Cyanidin-3-O-glucoside chloride, cyanidin 3-rutinoside chloride and peonidin 3-glucoside chloride, used as authentic standards for anthocyanin identification and for calibration curves preparation, were from Extrasynthese (Genay Cedex, France).

The Spectrum^TM^ Plant Total RNA kit was purchased from Merk Life Science S.r.l., (Milan, Italy) and the reagents for RT-qPCR analyses were all from Thermo Fisher Scientific (Waltham, MA, USA).

### 2.3. Anthocyanins Quantification by High Performance Liquid Chromatography Coupled with Diode Array Detection (HPLC-DAD)

The frozen samples were crushed using liquid nitrogen to obtain a fine powder. Extraction of anthocyanins was performed by adding 10 mL of pure methanol for each gram of powdered plant material and by shaking the samples in methanol for 24 h in the dark, at room temperature. After centrifugation at 10,000× *g* for 10 min, 2 mL of the supernatant was collected and stored at −20 °C until the analysis. All anthocyanins were separated and quantified by high-performance liquid chromatography (HPLC) using a Waters 1525 instrument equipped with a diode array detector (DAD) and a reversed-phase column (RP18 250 × 4.6 mm, 5 μM) with a pre-column (Phenomenex, Castel Maggiore, BO, Italy), as reported in [15]. Total anthocyanin quantification was performed at 520 nm using an external calibration curve with cyanidin-3-O-glucoside chloride as standard. 

The Phenol-Explorer database (http://phenol-explorer.eu/, accessed on 20 January 2023) was accessed online on March 20, 2023, to search for cyanidin-3-O-rutinoside (C3R) abundance and concentration in “fruits and fruit products” and “vegetables” categories. 

### 2.4. Anthocyanin Identification by High Performance Liquid Chromatography-Tandem Mass Spectrometry (HPLC-MS/MS)

Anthocyanins were identified by a liquid chromatography system HP 1290 Infinity Series equipped with a binary pump, a thermostated column compartment and a thermostated autosampler. The HPLC system was coupled with a 6420 Triple Quadrupole mass spectrometer, and both were from Agilent Technologies (Santa Clara, CA, USA). Experiments were carried out on selected sample extracts, choosing full scan and product ion as scan types. The following MS conditions were adopted throughout the trials: type of source: atmospheric pressure ionization-electrospray source (API-ES); polarity: positive; drying gas (nitrogen) temperature: 350 °C; gas flow: 13 L/min; nebulizer pressure: 50 psi; capillary voltage: +3500 V; collision gas: nitrogen; cell accelerator voltage: 3 V; collision energy: 25 eV; fragmentor: 80; and mass range: 250–1000 *m*/*z*. Compound separation was carried out on a C-18 column (Poroshell 120, SB-C18, 3.0 × 100 mm, 2.7 µm from Agilent Technologies, Palo Alto, CA, USA), using a flow rate of 0.8 mL min^− 1^, an injection volume and a column temperature set at 5 μL and 35 °C, respectively. The mobile phases consisted of 5% formic acid in milliQ water (A), and acetonitrile (B). The following multistep linear gradient was applied: 0 min, 5% B; 2 min, 7% B; 4 min, 9% B; 6 min, 12% B; 8 min, 15% B; 9 min, 16% B; 10 min, 17% B; 11 min, 17.5% B; 12 min, 18% B; 13 min, 100% B; 17 min, 100% B; 18 min, 5% B. The initial conditions were maintained for 5 min. Data were processed by the software MassHunter Workstation Software–Qualitative analysis (ver. B.06.00) from Agilent.

### 2.5. Bioinformatic Identification of Anthocyanin Encoding Genes and Phylogenetic Analysis

To identify *C. sativa* orthologs of the anthocyanin *MATE type transporter DETOXIFICATION 35* (*DTX35*) and *WD40 type TRANSPARENT TESTA GLABRA1* (*TTG1*), the *C. sativa* genome assembly cs10 was searched for using known anthocyanin encoding genes from other species as the query in NCBI, using the tblastn and blastp tools (https://blast.ncbi.nlm.nih.gov/Blast.cgi, accessed on 1 September 2022), as described in [13]. Query sequences are listed in Supplementary Table S2. Candidate sequences for MATE type transporters were selected based on their best hit score and used for estimating phylogenetic relationships using MEGA version XI package [16] (accessed on 1 September 2022). Full-length amino acid sequences were aligned by MUSCLE algorithm in the MEGA with default settings. Evolutionary relationships among sequences were inferred by using the neighbor-joining method [17] based on the *p*-distance method as described in [13]. The reliability of the phylogenetic tree was estimated by setting 1000 bootstrap replicates. The final figure of NJ trees was obtained by using the iTOL tree editor [18] (accessed on 5 September 2022).

### 2.6. RNA Isolation and RT-qPCR Analysis

Total RNA was isolated from 100 mg of plant material, using the Spectrum^TM^ Plant Total RNA kit. Two hundred nanograms of RNA were retrotranscribed using the High-Capacity RNA to cDNA kit for cDNA synthesis. Real-time polymerase chain reaction was performed with the Rotorgene 6000 (Corbett) using the PowerUp^TM^ Sybr^TM^ Green Master Mix as described previously [19]. Primers for RT-qPCR analysis (Table S1) were designed using the Primer3 Plus software (https://www.primer3plus.com/, accessed on 10 June 2022) [20,21] based on both already known (*CsF3′H, CsF3′5′H, CsDFR, CsANS, Cs3GT, CsOMT, CsMYB82, CsMYB87, CsbHLH112, CsbHLH114*)[13] and newly identified (*CsDTX35* and *CsTTG1)* sequences. The relative expression levels of the target genes were normalized using the reference genes *CsGAPDH (C. sativa Glyceraldehyde 3-phosphate dehydrogenase)* and *CsClat (C. sativa Clathrin).* The relative quantification of target gene expression was performed according to [22], and the mean of the fold change (n = 3) was reported as a binary logarithm, with the standard error of the mean. Pairwise comparison between the transcript levels of green and purple samples for each tissue and gene was done using Student’s *t*-test.

The correlation of the transcriptional levels between biosynthetic genes and transcription factors was investigated using the R-package corrplot (https://github.com/taiyun/corrplot) [23]. 

## 3. Results and Discussion

### 3.1. Evaluation of Anthocyanin Phenotype in C. sativa

**Figure 1:**
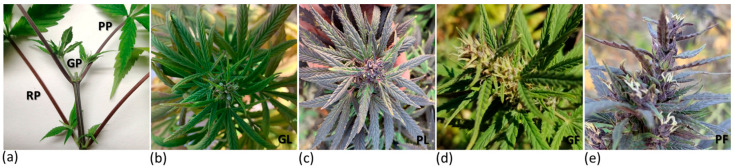
Representative photos of *C. sativa* samples used for molecular and biochemical analyses with various intensities of pigmentation. Green (GP), purple (PP) and red petioles (RP) (**a**) from Fibrante; green leaves (GL) (**b**), purple leaves (PL) (**c**), green female flower (GF) (**d**), and purple female flower (PF) (**e**) from accession S1750.

Starting from a germplasm collection composed of varieties, landraces, advanced selections and crossbreed populations, five *C. sativa* accessions were selected for the phenotype of interest, which was the accumulation of anthocyanins. A wide variation in pigmentation was observed among the selected accessions cultivated in the same site, including in the stage of development of appearance of the coloration, the affected tissues and the color itself. Therefore, different tissues (petioles, mature leaves and inflorescences), both green and pigmented, were collected from the diverse genotypes to compare anthocyanin accumulation both at biochemical and molecular level. Petioles of accession V18 were red throughout the life cycle of the plants, while in plants of accession S1652 and of the Fibrante variety, colors turning from green to red or purple were differently observed, even within the same plant. Petioles of Fibrante started to turn reddish or purple when the seedlings had about five pairs of true leaves. From this stage onward, the emerging petioles rapidly turned colored (Figure 1a). 

During the flowering stage, the plants of accession S1750 showed either presence or absence of pigmentation in leaves and flowers. However, by the end of the season, from mid-September to the end of October, all plants of this line exhibited purple pigmentation at the stem, petioles, leaves, and inflorescences (Figure 1b,c).

Plants of genotype S1759 showed red pigmentation only of the petioles and stem in vegetative stages, while at the end of the cycle the whole plant was completely red, including inflorescences and leaves.

Several plants (both male and female) from the PurpleF2 population were considered for their pigmentation. In fact, different individuals began to develop apical corolla coloration as early as at the very first vegetative stages, when the plant was about one month old. The tissues examined in this work consisted of pigmented inflorescences and their green counterpart.

### 3.2. Characterization and Quantification of Anthocyanins in C. sativa Tissues via HPLC-MS/MS

A total of four anthocyanins were identified in the different tissue samples through the combined information provided by elution order on the reversed phase column, co-chromatography with standards and UV–vis and mass spectra compared to the literature data [24,25].

Table 2 shows the UV and MS data (Supplementary Figure S1) of the anthocyanins identified in *C. sativa* tissues. All the identified compounds showed a typical UV maximum absorption between 499 and 523 nm and other maximum absorption in the range 275−280 nm. According to the literature [26], among others, these typical absorptions can be attributed to anthocyanin compounds, also confirmed by the following MS data. The first peak (peak 1), at 16.3 min, showed a molecular ion at *m*/*z* 449 and a fragment ion with 287 u ([M-162]^+^), corresponding to the cyanidin moiety as a result of a hexose molecule loss (−162 u) and in agreement with previous research [27]. The sugar moiety was confirmed to be glucose by co-elution of peak 1 with the cyanidin 3-glucoside standard (C3G). Indeed, red cyanidin glycosides are mostly found in vegetative tissues [28]. Peak 2 at 17.9 had a mass for the molecular ion at *m*/*z* 595. Accordingly, a MS2 fragmentation analysis was performed, and two transitions were observed in ions at *m*/*z* 287 and 449; the first one corresponded to the aglycon cyanidin, as a result of the loss of rutinoside (−308 u) and the fragment at *m*/*z* 449 resulted from the loss of a deoxyhexose ([M-146]^+^). This fragmentation pattern is in accordance with the literature [25,29] and it was also verified with the authentic standard. The anthocyanin was identified as cyanidin 3-rutinoside (C3R, also known as keracyanin). Peak 3, at 21.2 min, reported a parent ion at *m*/*z* 463 and a MS2 fragment at *m*/*z* 301, corresponding to the aglycone peonidin for the loss of a hexose unity (−162 u), as reported in [30,31]. This anthocyanin was identified as peonidin 3-glucoside (P3G) by its HPLC co-elution with the standard. Peak 4, at 23.1 min, presented the [M]^+^ at *m*/*z* 609 and MS2 fragments at *m*/*z* 463 and 301. According to [29,32] this compound was tentatively identified as peonidin 3-rutinoside (P3R). 

In the analysed samples, the total content of anthocyanins (TCA) ranged from a maximum of 1187.27 mg/100 g FW in purple leaf (PL) to a minimum of 3.00 mg/100 g FW in green leaf (GL) of the S1750 genotype (Table 3). No anthocyanins were detected in 4 out of the 16 samples and in particular in Fibrante, S1652 and PurpleF2 green petioles and flowers. Only di-substituted anthocyanins were detected by HPLC analyses and, among them, rutinose-conjugates anthocyanins were the prevailing form. Keracyanin (C3R) was found as the main anthocyanin characterizing *C. sativa* L., being always present in the highest concentration and, interestingly, the only one detected in reddish samples. In S1750 leaves and flowers, in S1759 flowers and in S1652 petioles P3R was also found with a concentration ranging from 2 to 20% of total anthocyanins. Glucose-conjugates of cyanidin and peonidin were found only in leaves and flowers of S1750, S1759, S1652 and Purple F2 which indeed showed a towards-purplish pigmentation and were among the samples with the highest concentration of anthocyanins.

Our results in general agree with those reported on Chinese cabbages leaves, in which TCA ranged from 20 to 60 mg/100 g FW [33] and in pigmented maize leaves (from 42.37 to 301.858 mg/100 g FW, [34]). Unexpectedly, concentrations of both keracyanin (885.58 mg/ 100 g FW) and TCA (1187.27 mg/100 g FW) in the pigmented tissues of *C. sativa* accession S1750 were by far higher than those of R. nigrum berries that, according to the Phenol Explorer database [35], have the highest abundance of C3R of the whole fruit and fruit-product category, corresponding to 160.78 mg/100 g FW with a TCA of 225.04 mg/100 g FW. 

Keracyanin possesses documented antihyperlipidemic, chemopreventive and chemotherapeutic activities [36,37,38,39]; thus the data here presented are of outstanding relevance for further exploitations of *C. sativa*-derived anthocyanins for several industrial and pharmacological applications. 

Considering that *C. sativa* is a fast-growing plant and that leaves and inflorescences are often wasted as by-products of fiber and seed production, the results presented here highlight the great potential of recovering these wastes and transforming them into high-added-value biochemicals following a circular economy approach. 

### 3.3. In Silico Identification of MATE Type and TTG1 Proteins in C. sativa

To date there is still a debate on how anthocyanins are transported into the plant cell vacuole and no flavonoid transporter has been characterized in *C. sativa* yet. Among the different intracellular systems (for a review [40]), the Multidrug and Toxic compound Extrusion (MATE) family of membrane transporters are known to mediate the trafficking of anthocyanins from the endoplasmic reticulum (ER) to the vacuole of plant cells [41] and proteins of MATE group II are known to be involved specifically in phenylpropanoid transport [42]. With the aim of identifying MATE type members in *C. sativa*, the cs10 proteome was searched using as the query the HMM signature (Pfam PF01554.19) and the blastp tool available in NCBI BLAST platform. 

**Figure 2:**
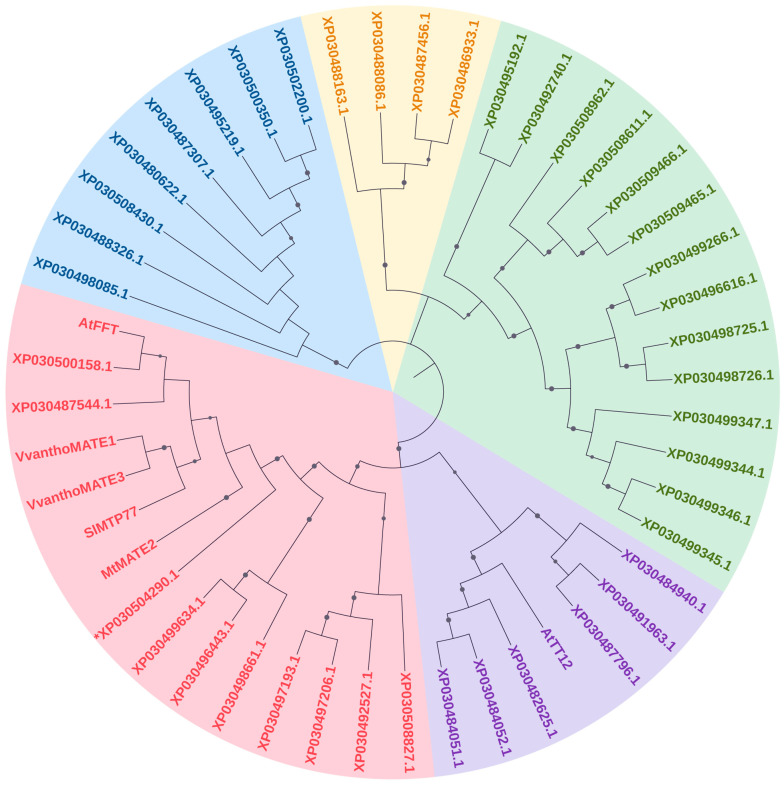
Genome wide phylogenetic tree of the putative *C. sativa* MATE proteins. Different colors indicate major phylogenetic subgroups. Red and purple subgroups belong to the group/type II of MATE transporters according to [42] while the others are *C. sativa* MATE transporters. Previously described flavonoid transporters belonging to MATEs were included: *Arabidopsis thaliana* FFT (AT4G25640.2) and AtTT12 (AT3G59030.1); *Vitis vinifera* VvanthoMATE1, (NP_001290007.1) and VvanthoMATE3 (NP_001268037.1); *Medicago truncatula* MtMATE2, (XP_003592215.2); *Solanum lycopersicum* MTP77 (Solyc03g025220.2.1). The evolutionary history was inferred using the Neighbor-Joining method [16]. The optimal tree is shown. The evolutionary distances were computed using the p-distance method and are in the units of the number of amino acid differences per site. This analysis involved 48 amino acid sequences. All ambiguous positions were removed for each sequence pair (pairwise deletion option). There were a total of 742 positions in the final dataset. Evolutionary analyses were conducted in MEGA11 [15]. The asterisk indicates the CsDTX35 (XP030504290.1) transporter. All amino acid sequences are reported in Supplementary Table S2. Bootstrap support values ≥ of 30% were indicated by grey circle on the tree branches.

A total of 43 putative MATE type proteins were retrieved and, to gain insights into their orthological relationships with known anthocyanin transport proteins from other species, a neighbor-joining phylogenetic analysis was performed. Three *C. sativa* proteins were grouped with already identified MATE type II members (Figure 2, the red clade) characterized by a high degree of similarity. We combined this result with a targeted blastp using as the query the amino acid sequence of tomato MTP77, which, according to its best hit score, allowed us to select for further analysis at transcriptional level the protein sequence XP_030504290.1, already annotated as *C. sativa* DETOXIFICATION 35 (CsDTX35).

The amino acid sequence of *S. lycopersicum* ANTHOCYANINS 11 (SlAN11), a WD40 repeat protein known to be involved in the regulatory MBW complex in tomatoes [4,43], was used as the query to retrieve the *C. sativa* ortholog sequence via the tblastn tool in NCBI. The gene locus LOC115716803 encoding for a WD-R containing protein and annotated as TRANSPARENT TESTA GLABRA 1 (TTG1) had the best hit score and thus was selected for transcript profiling in selected tissues.

### 3.4. Expression Profile of Structural Genes Controlling Anthocyanins Synthesis in Different Tissues

**Figure 3:**
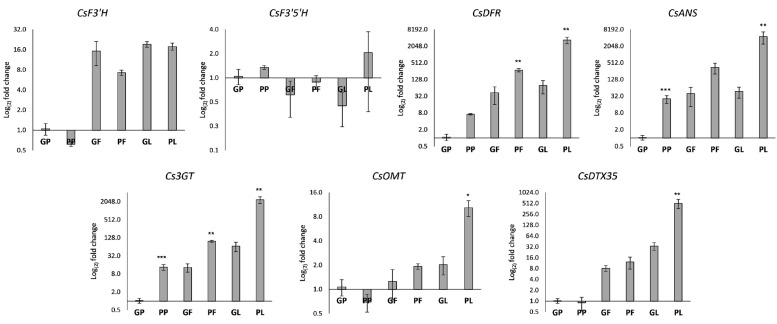
Relative expression levels of genes encoding late enzymes in different tissues of *C. sativa* by RT-qPCR. Transcription levels were calculated with the ΔΔCt method according to Pfaffl et al., 2005 using the Green Petiole (GP) as calibrator. The data are reported on a Log2 scale as the mean ± standard error of the mean (n = 3). Asterisks indicate Student’s *t*-test statistically significant differences: * *p* < 0.05; ** *p* < 0.01; *** *p* < 0.001. GP: Green petiole; PP: Purple petiole; GF: Green flower; PF: Purple flower; GL: Green leaf: PL: Purple leaf. GP and PP were from Fibrante; GF, PF, GL, and PL from s1750 accession. The whole name of analyzed Cannabis genes is detailed in the abbreviation list.

The chemical analysis evidenced that *C. sativa* tissues accumulated disubstituted cyanidin-based anthocyanins that are the major determinants of the red to purple phenotype not in petioles, leaves and inflorescences (Table 3). FLAVANONE 3′-HYDROXYLASE (F3′H) and FLAVANONE 3′,5′-HYDROXYLASE (F3′5′H) are alternative enzymes utilizing naringenin flavanone as substrate to produce dihydroxylated and trihydroxylated anthocyanidins, respectively. As shown in Figure 3, the CsF3′H gene was much more expressed than the CsF3′5′H gene in *C. sativa* samples. These results suggest that predominant transcription of F3’H compared to F3’5’H might stimulate the consumption of the precursor dihydrokaempferol for enhanced dihydroquercetin synthesis, thereby leading to the synthesis of more cyanidin-based anthocyanins, as was confirmed by biochemical analyses. 

Additional late enzyme-encoding genes were selected to address anthocyanin synthesis in *C. sativa* tissues. The DIHYDROFLAVONOL 4-REDUCTASE (DFR) and the ANTHOCYANIDIN SYNTHASE (ANS) are a key entry point in the synthesis of anthocyanin pigments, involved into the catalytic conversion of colorless dihydroflavonols to unstable anthocyanidins which are in turn subjected to decoration steps to become stable anthocyanins. Interestingly, on a plant species base, DFR can utilize diverse dihydroflavonols as substrate thus determining the anthocyanidin’s composition [44]. The *C. sativa* DFR and ANS encoding genes were previously identified in silico [13]. The expression analysis revealed that in anthocyanin-rich tissues the biosynthetic machinery is fully activated due to the induction of both CsDFR/CsANS (Figure 3), independently from the genotype. The transcription of the two genes is comparable: it is almost absent in green petioles, while it is induced in red petioles. As for leaves and inflorescences, a significant induction is observed in pigmented tissues, especially in leaves (*p* < 0.01).

Transcripts of the late enzyme-encoding genes were not found to be exclusive to red tissues but were also abundant in tissues described as green. This agrees with previous studies in tomatoes [45] that highlighted that regulatory proteins are required for the induction of the anthocyanin machinery in red organs and tissues. Indeed, a small but consistent accumulation of anthocyanins was also detected in the green tissues of the S1750 genotype, with C3R and P3R found in the leaves and both C3R and C3G in the inflorescences. The accumulation of these compounds is two orders of magnitude lower than in the pigmented tissues (PL and PF); however, it is enough to find that the structural genes involved in their synthesis were activated.

Based on the evidence of biochemical analysis, it was hypothesized that the glycosylation of cyanidin occurs via the involvement of the UDP-3-O-GLUCOSYLTRANSFERASES (3GT), thus leading to the synthesis of cyanidin-3-O-glucoside (C3G). In C3G-accumulating tissues, the transcript of Cs3GT is significantly upregulated (Figure 3), with increasing magnitude, proportional to the total C3G content. A similar step could be ascribed for the synthesis of cyanidin-3-O-rutinoside.

The biochemical data also showed the presence of peonidin-3-O-glucoside (P3G) in purple-colored tissue. It is well established that the enzyme flavonoid-3-O-methyltransferase (3-OMT) is involved in the synthesis of O-methylated anthocyanins, thus the transcription of a previously identified CsOMT was analyzed in pigmented tissues. Interestingly, the CsOMT transcriptional profile is remarkable in P3G (also known as methylcyanidin) accumulating tissues, suggesting that this enzymatic route can be responsible for anthocyanin synthesis in *C. sativa*. Once glycosylated, anthocyanins are translocated to the plant cell vacuole through different, not well-established mechanisms. The putative CsanthoMATE type transporter identified in silico, CsDTX35, was found to be transcribed more in purple leaves where TCA content was found to be the highest, and its transcriptional profile looks like those of CsOMT and Cs3GT involved in decoration steps (Figure 3). 

### 3.5. Transcription Factors Related to Anthocyanin Biosynthesis

The expression pattern of *C. sativa* genes encoding for the anthocyanin regulatory transcription factors MYB and bHLH was also evaluated in the same tissues.

Two CsMYB transcription factors (MYB33 and MYB78) were recently suggested to be responsible for the anthocyanin’s phenotype in a purple *C. sativa* accession [46]. These putative transcription factors were already annotated in [13] as *CsMYB82* and *CsMYB87,* respectively, belonging to subgroup 6 of R2R3-MYB; therefore, these gene names were used in this work. The results of our RT-qPCR analyses showed that both *CsMYB82* and *CsMYB87* were transcribed in all tissues and their transcriptional profiles were similar to those of *CsDFR* and *CsANS*, strongly supporting a role as master regulators of anthocyanin synthesis in *C. sativa*. 

CsMYB82, ortholog of the RUBY R2R3-MYB encoding gene known as the master regulator of red phenotype in Citrus [47], had been already proposed as the candidate master regulator for anthocyanin synthesis [46]. In our dataset, while in pigmented leaves it was transcriptionally active, in the purple inflorescences the gene was significantly less transcribed, probably due to the late date of sampling, when the regulator, having already fulfilled its function as a promoter of synthesis, was no longer intensely transcribed. CsMYB87, ortholog of Ipomea spp. MYB1 [48], was highly transcribed in pigmented leaves and inflorescences, strongly suggesting its regulatory role in promoting anthocyanin biosynthesis in these tissues. 

The expression of AN1 (CsbHLH112) and JAF13 (CsbHLH114) *C. sativa* orthologs was also tested. The transcriptional level of CsbHLH114 and CsMYB87 were similar in pigmented leaves suggesting that a bHLH114-MYB87 complex could be required to tightly regulate anthocyanins in *C. sativa* tissues.

**Figure 4:**
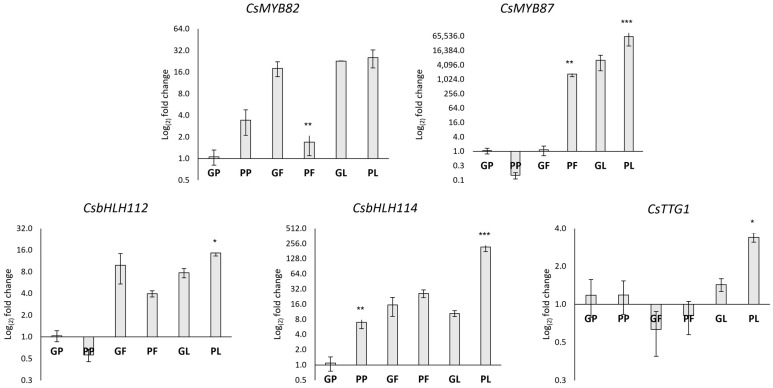
Relative gene expression levels of transcription factors in different tissues of *C. sativa* by RT-qPCR. Transcription levels were calculated with the ΔΔCt method according to Pfaffl et al. 2005 using the Green Petiole (GP) as calibrator. The data are reported on a Log2 scale as the mean ± standard error of the mean (n = 3). Asterisks indicate Student’s *t*-test statistically significant differences: * *p* < 0.05; ** *p* < 0.01; *** *p* < 0.001. GP: Green petiole; PP: Purple petiole; GF: Green flower; PF: Purple flower; GL: Green leaf: PL: Purple leaf. GP and PP were from Fibrante; GF, PF, GL, and PL from s1750 accession. The name of analyzed Cannabis genes is detailed in abbreviation list.

In addition, Figure 4 shows that the CsTTG1 gene is transcribed in all tissues; however a significantly higher abundance of the transcript is evident in pigmented leaves than in the other tissues. 

### 3.6. Correlation Analysis of the Expression Profiles of Anthocyanin Genes

**Figure 5:**
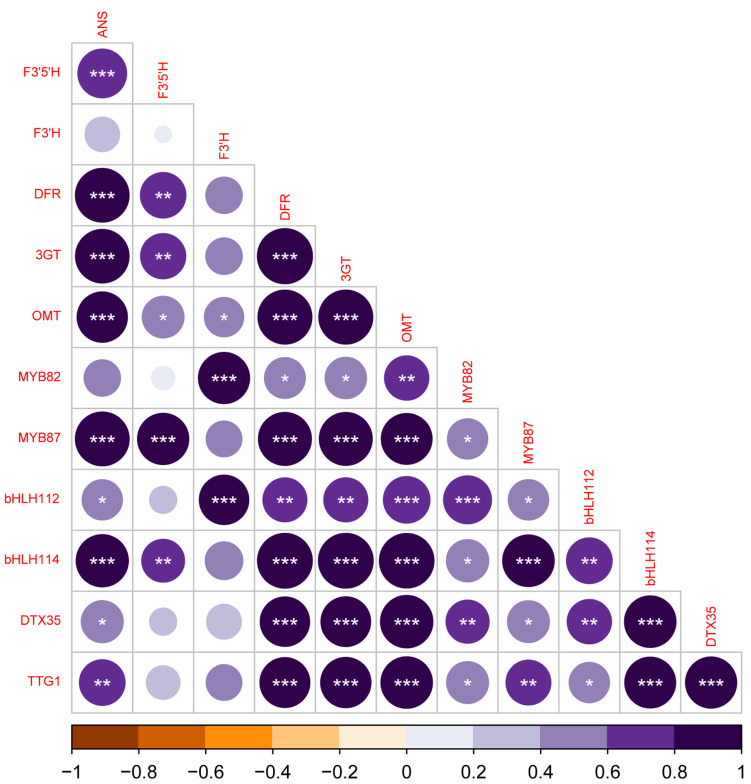
Correlation plot of Cannabis anthocyanin-related structural and regulatory genes. Correlation analysis using Pearson’s correlation matrix of transcriptional level of structural and regulatory genes involved in the anthocyanin synthesis. Dark purple indicates positive correlation, brown indicates negative correlation. Asterisks denote significant association between samples (*, *p* < 0.05; **, *p* <0.01; ***, *p* < 0.001).

A correlation analysis was performed to assess the potential correlations between structural and regulatory genes. As shown in Figure 5, the expression of CsMYB87 correlates with several genes encoding for anthocyanin-related enzymes, a lower number of structural genes showed a correlation with the expression of CsMYB82. Interestingly, the two genes encoding for F3′H and F3′5′H show a different pattern of correlation with CsMYB82 and CsMYB87, respectively. Regarding the bHLH class of TF, both CsbHLH112 and CsbHLH114 showed a correlation with the expression of several structural genes and, interestingly, with CsMYB82 and CsMYB87, respectively. Furthermore, even the expression of both the MATE type transporter encoding gene and CsTTG1 correlates with several candidate structural and regulatory genes.

**Figure 6:**
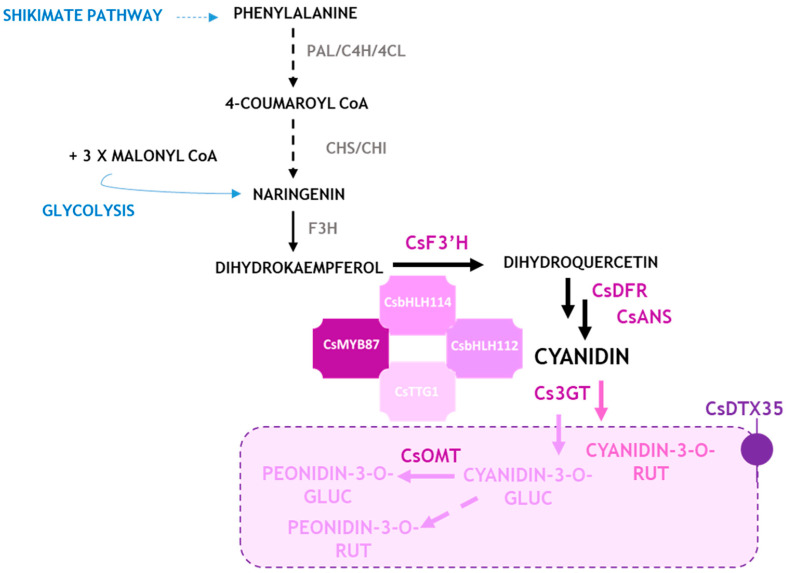
Proposed model for anthocyanin synthesis and regulation in *C. sativa*. For enzymes, please refer to the abbreviation list. The dotted arrows and enzymes written in grey stand for hypothesized steps and/or not analyzed in this work, respectively.

Although the nature and stoichiometric composition of MBW complexes in Cannabis is yet to be elucidated through functional approaches, taken together these results allowed us to propose a model of anthocyanin synthesis and regulation in Cannabis (Figure 6).

## 4. Conclusions

Our work identified keracyanin for the first time as the major anthocyanin in *Cannabis sativa*, casting light on the exploitation of these less investigated metabolites in a circular economy scenario. Of great interest, the amount of this bioactive compound was found to be higher than in the other natural sources known to date. This result, along with the fact that this species is fast-growing and develops a great biomass, makes *C. sativa* exploitable as an important source of this bioactive metabolite. Furthermore, the identification of the molecular players controlling anthocyanin synthesis and regulation will allow researchers to boost both breeding and metabolic engineering strategies for anthocyanin-specific accumulation in plant tissues.

## Figures and Tables

**Table 1:** List of *C. sativa* L. genotypes and collected samples. Sampling time is indicated as days after sowing (das). Growth stage is indicated according to the BBCH stage [14] and briefly described. All samples were taken in three biological replicates (n = 3).

Genotype	Plant Tissue	Color	Sampling Time	Growth Stage
Fibrante	petiole	green	37	BBCH 14 (4 true leaf pairs)
Fibrante	petiole	purple	53	BBCH 18 (8 true leaf pairs)
S1750	female flower	green	164	BBCH 67 (flowering finishing)
S1750	female flower	purple	164	BBCH 67 (flowering finishing)
S1750	leaves	purple	164	BBCH 67 (flowering finishing)
S1750	leaves	green	164	BBCH 67 (flowering finishing)
S1759	flowers	purple	164	BBCH 67 (flowering finishing)
S1652	petiole	green	53	BBCH 18 (8 true leaf pairs)
S1652	petiole	purple	53	BBCH 18 (8 true leaf pairs)
PurpleF2	female flower	green	118	BBCH 65 (full flowering)
PurpleF2	female flower	purple	118	BBCH 65 (full flowering)
PurpleF2	male flower	purple	118	BBCH 60 (first individual flowers open)
V18	petiole	red	164	BBCH 67 (flowering finishing)

**Table 2:** Chromatographic, UV–vis and mass spectroscopy characteristics of the anthocyanins from *C. sativa*, obtained by HPLC–DAD and HPLC-MS/MS.

Peak	t_R_ (min)	λ Max (nm)	[M]^+^ (*m*/*z*)	MS/MS (*m*/*z*)	Confirmed by Standard	Compounds
1	16.3	280, 519	449	287	+	Cyanidin 3-glucoside
2	17.9	280, 522	595	449, 287	+	Cyanidin 3-rutinoside
3	21.1	275, 519	463	301	+	Peonidin 3-glucoside
4	23.1	280, 523	609	463, 301	−	Peonidin 3-rutinoside

**Table 3:** Main anthocyanins found in *C. sativa* L. genotypes. G/P/R P, green/purple/red petiole; P/G L, purple/green leaf; G/P F, green/purple inflorescence; C3G, cyanidin-3-O-glucoside; C3R, cyanidin 3-rutinoside; P3G, peonidin 3-glucoside; P3R, peonidin 3-rutinoside. TCA, total content of anthocyanins. Anthocyanins are expressed as mg/100 g Fresh Weight (FW).

Genotype	Plant Material	C3G	C3R	P3G	P3R	TCA
Fibrante	GP	0.00	0.00	0.00	0.00	0.00
Fibrante	GP	0.00	0.00	0.00	0.00	0.00
Fibrante	PP	0.00	29.38	0.00	0.00	29.38
Fibrante	PP	0.00	15.26	0.00	0.00	15.26
Fibrante	PP	0.00	18.07	0.00	0.00	18.07
S1750	GF	0.56	4.43	0.00	0.00	4.99
S1750	PF	6.48	312.53	4.66	36.47	360.13
S1750	PL	19.88	885.58	32.48	249.33	1.187.27
S1750	GL	0.00	2.35	0.00	0.65	3.00
S1759	PF	0.00	31.09	0.00	0.91	32.00
S1652	GP	0.00	0.00	0.00	0.00	0.00
S1652	PP	0.52	168.49	0.78	1.75	171.54
PurpleF2	GF	0.00	0.00	0.00	0.00	0.00
PurpleF2	PF	0.00	9.64	0.00	0.00	9.64
PurpleF2	* MPF	0.58	108.72	0.63	0.00	109.93
V18	** RP	0.00	11.23	0.00	0.00	11.23

* Male Purple Flower; ** Red Petiole.

## Data Availability

Data is contained within the article and Supplementary Materials.

## Supplementary Materials

The following supporting information can be downloaded at: https://www.mdpi.com/article/10.3390/antiox12071393/s1, Figure S1. Mass spectra of the four identified peaks in Cannabis samples; Table S1: List of primer pairs used for RT-qPCR; Table S2. List of amino acid sequences used for phylogenetic analysis in Figure 2.

## Abbreviations

ROS	reactive oxygen species
C3R	cyanidin-3-rutinoside or keracyanin
P3R	peonidin-3-rutinoside
C3G	cyanidin-3-glucoside
P3G	peonidin-3-glucoside
TCA	total content of anthocyanin
MYB	myeloblastosis
CsMYB82	*Cannabis sativa* myeloblastosis82
CsMYB87	*Cannabis sativa* myeloblastosis87
bHLH	basic helix loop helix
CsbHLH112	*Cannabis sativa* basic helix loop helix 112
CsbHLH114	*Cannabis sativa* basic helix loop helix 114
3GT	UDP-GLUCOSE:FLAVONOID 3-O-GLUCOSYLTRANSFERASE
DTX35	MATE type trans-porter DETOXIFICATION 35
WD40	tryptophan-aspartic acid repeat domains
TTG1	WD40 containing type TRANSPARENT TESTA GLABRA1
AN1	Anthocyanin 1
JAF13	JOHN AND FRANCESCA 13
PAL	phenylalanine ammonia lyase;
4CL	4-coumarate-CoA ligase;
C4H	P450 monooxygenase cinnamate-4-hydroxylase;
CHS	chalcone synthase;
CHI	chalcone isomerase;
F3H	flavanone 3-hydroxylase;
F3′H	flavonoid 3′-hydroxylase;
F3′5′H	flavonoid 3′,5′-hydroxylase;
DFR	dihydroflavonol 4-reductase;
ANS	anthocyanidin synthase;
3-OMT	flavonoid-3-O-methyltransferase;
MATE	multi-antimicrobial extrusion protein
MBW	MYB-bHLH-WD40 complex

## References

[1] Saigo T., Wang T., Watanabe M., Tohge T. (2020). Diversity of anthocyanin and proanthocyanin biosynthesis in land plants. Curr. Opin. Plant Biol..

[2] Wong D.C.J., Pichersky E., Peakall R. (2023). Many different flowers make a bouquet: Lessons from specialized metabolite diversity in plant-pollinator interactions. Curr. Opin. Plant Biol..

[3] Schaefer H.M., McGraw K., Catoni C. (2008). Birds use fruit colour as honest signal of dietary antioxidant rewards. Funct. Ecol..

[4] Kiferle C., Fantini E., Bassolino L., Povero G., Spelt C., Buti S., Giuliano G., Quattrocchio F., Koes R., Perata P. (2015). Tomato R2R3-MYB Proteins SlANT1 and SlAN2: Same Protein Activity, Different Roles. PLoS ONE.

[5] Kaur S., Tiwari V., Kumari A., Chaudhary E., Sharma A., Ali U., Garg M. (2023). Protective and defensive role of anthocyanins under plant abiotic and biotic stresses: An emerging application in sustainable agriculture. J. Biotechnol..

[6] Bassolino L., Petroni K., Polito A., Marinelli A., Azzini E., Ferrari M., Ficco D.B., Mazzucotelli E., Tondelli A., Fricano A. (2022). Does Plant Breeding for Antioxidant-Rich Foods Have an Impact on Human Health?. Antioxidants.

[7] Chun O.K., Kim D.O., Moon H.Y., Kang H.G., Lee C.Y. (2003). Contribution of individual polyphenolics to total antioxidant capacity of plums. J. Agric. Food Chem..

[8] Holton T.A., Cornish E.C. (1995). Genetics and Biochemistry of Anthocyanin Biosynthesis. Plant Cell.

[9] Winefield W., Davies K., Gould K., Winefield W., Davies K., Gould K. (2008). Anthocyanins.

[10] Koes R., Verweij W., Quattrocchio F. (2005). Flavonoids: A colorful model for the regulation and evolution of biochemical pathways. Trends Plant. Sci..

[11] Irani N.G., Hernandez J.M., Grotewold E., John T.R. (2003). Chapter three Regulation of anthocyanin pigmentation. Recent Advances in Phytochemistry.

[12] Zhang Y., Butelli E., Martin C. (2014). Engineering anthocyanin biosynthesis in plants. Curr. Opin. Plant Biol..

[13] Bassolino L., Buti M., Fulvio F., Pennesi A., Mandolino G., Milc J., Francia E., Paris R. (2020). In Silico Identification of MYB and bHLH Families Reveals Candidate Transcription Factors for Secondary Metabolic Pathways in *Cannabis sativa* L.. Plants.

[14] Mishchenko S., Mokher J., Laiko I., Burbulis N., Kyrychenko H., Dudukova S. (2017). Phenological growth stages of hemp (*Cannabis sativa* L.): Codification and description according to the BBCH scale. Žemės Ūkio Moksl..

[15] Mattivi F., Guzzon R., Vrhovsek U., Stefanini M., Velasco R. (2006). Metabolite profiling of grape: Flavonols and anthocyanins. J. Agric. Food Chem..

[16] Kumar S., Stecher G., Li M., Knyaz C., Tamura K. (2018). MEGA X: Molecular evolutionary genetics analysis across computing platforms. Mol. Biol. Evol..

[17] Tamura K., Peterson D., Peterson N., Stecher G., Nei M., Kumar S. (2011). MEGA5: Molecular evolutionary genetics analysis using maximum likelihood, evolutionary distance, and maximum parsimony methods. Mol. Biol. Evol..

[18] The iTOL Platform. https://itol.embl.de/.

[19] Fulvio F., Paris R., Montanari M., Citti C., Cilento V., Bassolino L., Moschella A., Alberti I., Pecchioni N., Cannazza G. (2021). Analysis of Sequence Variability and Transcriptional Profile of *Cannabinoid synthase* Genes in *Cannabis sativa* L. Chemotypes with a Focus on *Cannabichromenic acid synthase*. Plants.

[20] Koressaar T., Remm M. (2007). Enhancements and Modifications of Primer Design Program Primer3. Bioinformatics.

[21] Untergasser A., Cutcutache I., Koressaar T., Ye J., Faircloth B.C., Remm M., Rozen S.G. (2012). Primer3—New capabilities and interfaces. Nucleic Acids Res..

[22] Pfaffl M.W. (2001). A new mathematical model for relative quantification in real-time RT-PCR. Nucleic Acids Res..

[23] Wei T., Simko V. (2021). R Package ‘corrplot’: Visualization of a Correlation Matrix. (Version 0.92). https://github.com/taiyun/corrplot.

[24] Wu X., Prior R. (2004). Systematic identification and characterization of anthocyanins by HPLC–ESI–MS/MS in common foods in the United States: Fruits and berries. J. Agric. Food Chem..

[25] Gómez-Caravaca A.M., Verardo V., Toselli M., Segura-Carretero A., Fernández-Gutiérrez A., Caboni M.F. (2013). Determination of the major phenolic compounds in pomegranate juices by HPLC−DAD−ESI-MS. J. Agric. Food Chem..

[26] Downey M.O., Rochfort S. (2008). Simultaneous separation by reversed-phase high-performance liquid chromatography and mass spectral identification of anthocyanins and flavonols in Shiraz grape skin. J. Chromatogr. A.

[27] Olivas-Aguirre F.J., Rodrigo-García J., Martínez-Ruiz N.D.R., Cárdenas-Robles A.I., Mendoza-Díaz S.O., Álvarez-Parrilla E., González-Aguilar G.A., De la Rosa L.A., Ramos-Jiménez A., Wall-Medrano A. (2016). Cyanidin-3-O-glucoside: Physical-chemistry, foodomics and health effects. Molecules.

[28] Steyn W.J., Wand S.J.E., Holcroft D.M., Jacobs G. (2002). Anthocyanins in vegetative tissues: A proposed unified function in photoprotection. New Phytol..

[29] de Rosso V.V., Hillebrand S., Montilla E.C., Bobbio F.O., Winterhalter P., Mercadante A.Z. (2008). Determination of anthocyanins from acerola (*Malpighia emarginata* DC.) and acai (*Euterpe oleracea* Mart.) by HPLC–PDA–MS/MS. J. Food Compos. Anal..

[30] Castañeda-Ovando A., Sedo O., Havel J., Pacheco L., Galán-Vidal C.A., Contreras López E. (2012). Identification of anthocyanins in red grape, plum and capulin by MALDI-ToF MS. J. Mex. Chem. Soc..

[31] de Pascual-Teresa S., Santos-Buelga C., Rivas-Gonzalo J.C. (2002). LC–MS analysis of anthocyanins from purple corn cob. J. Agric. Food Chem..

[32] Pradhan P.C., Saha S. (2016). Anthocyanin profiling of Berberis lycium Royle berry and its bioactivity evaluation for its nutraceutical potential. J. Agric. Food Chem..

[33] He Q., Zhang Z., Zhang L. (2016). Anthocyanin accumulation, antioxidant ability and stability, and a transcriptional analysis of anthocyanin biosynthesis in purple heading Chinese cabbage (*Brassica rapa* L. ssp. *pekinensis*). J. Agric. Food Chem..

[34] Paulsmeyer M.N., Vermillion K.E., Juvik J.A. (2022). Assessing the diversity of anthocyanin composition in various tissues of purple corn (*Zea mays* L.). Phytochemistry.

[35] Neveu V., Perez-Jiménez J., Vos F., Crespy V., du Chaffaut L., Mennen L., Knox C., Eisner R., Cruz J., Wishart D. (2010). Phenol-Explorer: An online comprehensive database on polyphenol contents in foods. Database.

[36] Feng R., Ni H.M., Wang S.Y., Tourkova I.L., Shurin M.R., Harada H., Yin X.M. (2007). Cyanidin-3-rutinoside, a natural polyphenol antioxidant, selectively kills leukemic cells by induction of oxidative stress. J. Biol. Chem..

[37] Chen P.N., Chu S.C., Chiou H.L., Kuo W.H., Chiang C.L., Hsieh Y.S. (2006). Mulberry anthocyanins, cyanidin 3-rutinoside and cyanidin 3-glucoside, exhibited an inhibitory effect on the migration and invasion of a human lung cancer cell line. Cancer Lett..

[38] Thilavech T., Adisakwattana S. (2019). Cyanidin-3-rutinoside acts as a natural inhibitor of intestinal lipid digestion and absorption. BMC Complement. Altern. Med..

[39] Gonçalves A.C., Nunes A.R., Falcão A., Alves G., Silva L.R. (2021). Dietary Effects of Anthocyanins in Human Health: A Comprehensive Review. Pharmaceuticals.

[40] Zhao J. (2015). Flavonoid transport mechanisms: How to go, and with whom. Trends Plant. Sci..

[41] Biała W., Jasiński M. (2018). The Phenylpropanoid Case—It Is Transport That Matters. Front. Plant. Sci..

[42] Nimmy M.S., Kumar V., Suthanthiram B., Subbaraya U., Nagar R., Bharadwaj C., Jain P.K., Krishnamurthy P. (2022). A Systematic Phylogenomic Classification of the Multidrug and Toxic Compound Extrusion Transporter Gene Family in Plants. Front. Plant. Sci..

[43] Gao Y., Liu J., Chen Y., Tang H., Wang Y., He Y., Ou Y., Sun X., Wang S., Yao Y. (2018). Tomato SlAN11 regulates flavonoid biosynthesis and seed dormancy by interaction with bHLH proteins but not with MYB proteins. Hortic. Res..

[44] Grotewold E. (2006). The genetics and biochemistry of floral pigments. Annu. Rev. Plant Biol..

[45] Butelli E., Titta L., Giorgio M., Mock H.P., Matros A., Peterek S., Schijlen E.G., Hall R.D., Bovy A.G., Luo J. (2008). Enrichment of tomato fruit with health-promoting anthocyanins by expression of select transcription factors. Nat. Biotechnol..

[46] Kundan M., Gani U., Fayaz M., Angmo T., Kesari R., Rahul V.P., Gairola S., Misra P. (2022). Two R2R3-MYB transcription factors, CsMYB33 and CsMYB78 are involved in the regulation of anthocyanin biosynthesis in *Cannabis sativa* L.. Ind. Crops Prod..

[47] Butelli E., Licciardello C., Zhang Y., Liu J., Mackay S., Bailey P., Reforgiato-Recupero G., Martin C. (2012). Retrotransposons control fruit-specific, cold-dependent accumulation of anthocyanins in blood oranges. Plant Cell.

[48] Lu Y., Du J., Tang J., Wang F., Zhang J., Huang J., Liang W., Wang L. (2009). Environmental regulation of floral anthocyanin synthesis in Ipomoea purpurea. Mol. Ecol..

