# Supplementary material for: When Cannabis sativa L. Turns Purple: Biosynthesis and Accumulation of Anthocyanins
Source: Antioxidants (Basel). 2023 Jul 6;12(7):1393. doi: 10.3390/antiox12071393 (PMC10376404; doi:10.3390/antiox12071393)
Supplement: Supplementary file 1 [file antioxidants-12-01393-s001.zip › antioxidants-2427305-supplementary.pdf]

antioxidants

MDPI

Supplemenatry Materials

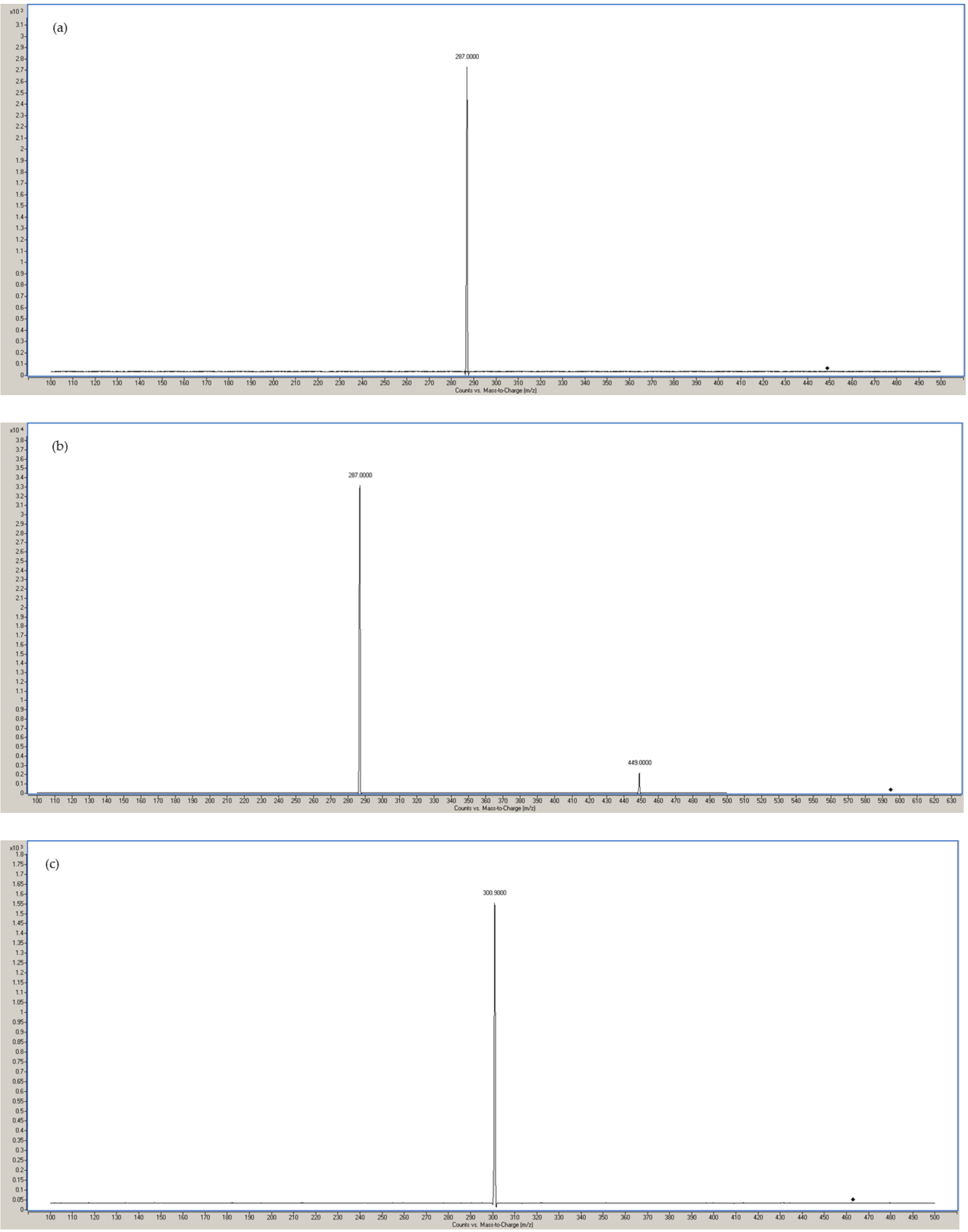

Figure S1. Mass spectra of the four identified peaks in *Cannabis* samples. Second-order mass spectra, obtained with ESI ionization in positive mode, of cyanidin 3-glucoside (peak 1), according to the standard mass spectra as reported by Olivas-Aguirre et al., 2016 (a), second-order mass spectra, obtained with ESI ionization in positive mode, of cyanidin 3-rutinoside (peak 2), according to the standard mass spectra as reported by de Rosso et al., 2008 (b), second-order mass spectra, obtained with ESI ionization in positive mode, of peonidin 3-glucoside (peak 3), according to the standard mass spectra as reported by Castañeda-Ovando et al. (2012) and De Pascual-Teresa et al. (2002)

(c),.second-order mass spectra, obtained with ESI ionization in positive mode, of peonidin 3-rutino-side (peak 4) according to the standard mass spectra as reported by de Rosso et al. (2008) and Pra-dhan and Saha (2016).

Table S1. List of primer pairs used for RT-Qpcr. Forward (Fw) and reverse (Rv) primer sequences, the accession number of cs10 transcript used for primer design, amplicon length expressed in base pairs (bp), PCR efficiency (Eff) are given.

Gene	NCBI transcript	Primer sequences	Effi	Amplicon length
CsF3'H	XM_030637390.1	FW:CGACACATCATCAAGCACAGT<br>RV:CAGCATCACTTACAAGCCTATCTC	0.85	125 bp
CsF3'5'H	XM_030644276.1	FW:ATTGCGTGTACATCCACCTG<br>RV:GGATTTTCCCAAAGGCTAGG	1.07	147 bp
CsANS	XM_030645652.1	FW: GCAATGTTTTCGAGGAGGAG<br>RV: TCAACTCCTCTCGGCATTTC	1.34	114 bp
CsDFR	XM_030638485.1	Fw:TTCTCCACCTCGGTTACA<br>Rv:TCAGCCTTCCACAGAGTA	1	118 bp
CsOMT	XM_030640905.1	Fw:ACACGAGCAGTTGAAGGAGC<br>Rv:TGTCCTTCATCCACAGGCAC	1.46	87 bp
CsDTX35	XM_030648430.1	Fw:CAATCTTAACGGGTGGGAAG<br>Rv:TTGGACGTTTCCTACCAAGC	1.64	92 bp
Cs3GT	XM_030645093.1	Fw:CCGAGATTGGGAAGGAGGTG<br>Rv:AAGGCCCAGCAGTCCAAAAT	1.54	114 bp
CsTTG1	XM_030645708.1	Fw:ATAGCTTGGGGTGAAGCAAG<br>Rv:AGCAAAGGGGTATCAGGTTG	1.18	128 bp
CsMYB82	XM_030636983.1	Fw:ACCAGGAAGGACAGCCAATG<br>Rv:AGGTCCGAGGACGAGGTTTA	1.21	167 bp
CsMYB87	XM_030622841.1	FW: CGTGGAGAATTTGAGGAGGAC<br>RV: TCTACCGGCAATCAATGACC	1.45	87 bp
CsbHLH112	XM_030646090.1	FW:GCCACTTCATCAGACAAC<br>RV: CTCCTCATCCTCTTCTTCAT	1.47	165 bp
CsbHlH114	XM_030630819.1	FW:GCCACTTCATCAGACAAC<br>RV:CTCCTCATCCTCTTCTTCAT	1.27	162 bp

Table S2. Amino acid sequences used for Neighbour-Joining phylogenetic analysis reported in Fig-ure 2.

>XP030500158.1
MAAEEAAAVPLLTENTGPEEEDKDYEAVVSVRDVRNVFWKESVKLWTIAGPIICTIVCNY
GINSLTNIFVGHIGELQLSAVTISLSVIGTFSFGFMLGMGSALETLCGQAFGAGQVHMLG
IYMQRSWLILLVSCFFLLPFYIFAAPILKILGQADDVADLAGKFTILVIPQLFSLAVNFP
TQKFLQAQSKVNALAWIGFVAMVLHVGLLWLFIYFFNWGLTGAAVAFDLTSWLIVIAQVV
YVICWCNEGWSGFSMLAFKELWAFVRLSLASAVMLCLEIWYLMSVIVLTGHLKNAVIAVD
SLSVCMNLNGWEAMLFIGINAAISVRVSNELGMGRPLGAKYSVYVTVFQSLLIGIFFAVV
ILLTKDDFSVIFTNSKEVQEAVSRLAYLLGITMLLNSVQPVISGVAIGGGWQALVAYINL
GCYYIFGLPLGVLLGYTAGLGVIGLWGGMICGTALQTLLLLIVLYKTNWNKEVEQTTERM
RLWAGQDVIEKPADQHI
>XP030487544.1
MPPESTITAIAFEIECVFSEWSKRNAKQTKEKGLALFLYYYHYNKTKSVKIGNKRKENEA
VLKLEEEEDGEERRRSSASSSSSSSSVLLSSTTLITSSSSSPSSPPPSYSKKGVNGFDGV
SILESAELQYAPTGVVGSTDDEDGDGDNKDYPAITNFHDAKTIFIVESSKLWTIACPIAF
NIWCNYGINSFTNIFVGHLGDVELSAVAISLSVIANFSFGFLLGMGSALETLCGQAFGAG
QVDMLGVYMQRSWIILFTACFFLLPLYIFSTPILKLLGQEDQIAELAGKFTIQIIPQMFS
LAINFPTQKFLQAQSKVTILAWVGFGALIMHTGILYLFIKVFDWGTTGAAAAYDISAWAV

AIAQVVYIVGGCQDGWKGLSWLAFKDMWAFVKLSVASAVMLCLEIWYFMTIIVLTGHLED
PIIAVGSLSICMNINGWEGMLFIGVNAAISVRVSNELGSGHPRAAKYTVIVTCVEALCIG
ILCAMLILLTKDHFAIIFTDSKEMQEAVSRLAFLLGITMVLNSVQPVISGVAVGGGWQAL
VAYINLFCYYLIGLPLGFFLGYRTNLKVEGIWIGMICGTFLQTLILVYIIYKTNWTKEVE
QASERLRGWTAQENGSDIIKV
>XP030504290.1
MDEPLLERTASEIVNGNINNHVSAEEGIINEDYSPARGFKELKTVFWLESLKLWRIAGPA
VVTIMCQYGTNSATSIFVGHLGDIQLSAISISLGVVGTFSFGFMLGMGSALETLCGQAFG
AGQVHMLGIYMQRSWIILFSFCILLTPIYIFAGPILKLLGQNDEIADLAGQFSIQIIPQL
FSLAFNFPAQKFLQSQSKFDVLAWISFLALIAHIGLLWLFIYVLSLGTMGAAIAFNITRW
GLCLAQIVYIMRWCNEGWTGFSWLAFKDLWAFLKLSFASALMLCLEIWYIMSIYLLTGSL
DNAVIAVGSLSICLNLNGWEGILFVGVNAAISVRVSNELGRKRPRAAKYSVYVTLFQALL
IGLFFMIVVLVARQYLALIYTNDAALRKSVSHLAYLLGFTMLLNSVQQVISGVAIGGGWQ
STVAYVNLGSYYIFGLPLGYVLGHYAGFGVMGLWGGMIIGIALQTLILSIILYRTNWTKE
VEQSTERVREWGGEDTNDKATQDQI
>XP030499634.1
MEENNNNQPLLSSREENQNNHLETLLSTTSNHSFTADADDIQPINTVRDFYKEFKVESKK
LWFLAGPAIFTSICQYSLGAITQVFAGHVSTIALAAVSVENSVIAGFSFGVMLGMGSALE
TLCGQAFGAGQHDMLGIYMQRSWVILNSTGVILCFLYIFASKLLKLIGQTTEISEAAGLF
AVWMIPQLFAYAMNFPIAKFLQAQSKMMVMAIISAVALVFHTFFSWLLILKLDWGMAGAA
VVLNVSWWFIVVAQLAYIFSGTCGRAWSGFSSKAFQNLWGFVRLSLASAVMLCLEVWYFM
ALILFAGYLKNAKISVDALSICMNILGWTVMVALGINAAISVRVSNELGAAHPRTAKFSL
VVAVISSFIIGLVLSIILIASRNEYPTLFSDDSDVETLVKKLTPVLATCILINNIQPVLS
GVAIGAGWQAAVAYVNIVCYYVFGVPLGLIMGYKLDMGVTGIWCGMLSGTVVQTCVLFFM
VYKTNWNKEASVAEDRIRKWGGPDSKEDNDV
>XP030498661.1
MEDNRNPLLHSLGGGHGSRPTSASFTSSFTADGDDIQPINDVPSFFKEFTIETKKLWYIA
GPAIFTSVCQYSLGAITQIFSGQLGTLELAAVSVQNFVISGFTFGVLLGMGSALETLCGQ
AFGAGQLNMLGIYMQRSWIVLFVTGLILSLFYIFAEPILLLIGQTPEISKATGLYCLYVL
PEIFAYALNFPITKFLQSQSKILVMSAIAAIALVLHTFFSWLFMLKLGWGLVGAAVVMNT
SWWFIVVAQFIYILSGTCGEAWSGFSWKAFHNLWGFVKLSFASAVMLCLELWYFMVLIVF
AGYLKNAQVSVDAMSICMNILDWTIMVSVGVNAAISVRVSNELGAAHPRTAKFSVVVAVI
TSFICGLLLALLLMVSRNQYPTIFSIDTEVQNVVKDLTPVLAFSIVINNIQPTLSGVAIG
AGWQAAVAYVNIACYYVFGIPLGLILGYKLNMGITGIWYGMLAGTVLQTAVIFWIVYRTN
WNKEASLAGDRIKKWGG
>XP030496443.1
MEKENNQPLLSPYGEDELTHQNKHLLSRPFTAGARLLFTEFKTEFNKLWYLAAPAIFTAI
CQYSLGAITQVFAGHVSTLALAAVSVENSVISGFSFGIMLGMGSALETLCGQAYGAGQYD
MLGIYMQRSWLILNGTAVILCFFYIFAEQLLKLIGQTPEISESAGLFSVWMIPQLFAYAL
NFPIAKFLQAQSKIMVMAVISAVALVFHVFFSWLVMLKLGWGMAGAAVVLNLSWWFIVVA
QLAYIFSGSCGRAWSGFSWKAFQNLWGFVKLSFASAIMLCLEIWYFMALVLFAGYLKNAE
VSVDVLSICANILGWAMMVAIGMNAAISVRVSNELGADHPRTAKVSLIVAVITSFLIGVV
LSLILVITRNQYPSLFSDDTSVRILVTNLTPLLATCIVINNIQPVLSGVAIGAGWQAFVA
YVNVACYYLFGVPLGLSLGYKLNMGVSGIWYGMLAGTVLQTSILFLMVYTTNWSKEASLA
EDRIRKWGGDSNEDIEKLVK
>XP030487796.1
MENSRRISSEQLLLQLHEPMLTMKSSPISSELEDTLSNPDIPFFQRLKSATSIELNTLFR
LAGPTVVVYLLNNVTSMSTQIFCGHLGNLQLAAASLGNTGIQVFAYGLMLGMGSAVETLC
GQAYGAHKYGMLGIYLQRSTILLMATGIPLTIIYAFSKPILLGLGESEEIASAAALFVYG
LIPQIFAYAANFPIQKFLQAQSIVFPSAYISAGTLVVHLVLSWVLIYKLGWGLIGASLML
SFSWWIVVVAQFVYILMSDKCKLTWNGFSLEAFSGLWEFFKLSIASAVMLCLETWYYQIL
VLIAGLLKNAEIALDSLSVCMTISGWVFMIAVGFNAAASVRVGNEIGAGHPKSASFAVVI
VTLSSFLISLVFAILVLVLRHVISYAFTDGVTVANAVSDLTPFLAVSIILNGIQPVLSGV
AVGCGWQAFVAYVNVGCYYIIGIPLGALLGFTFNLGAKGIWTGMICGTLIQTIILLWVTF
RTDWNKEVEKAKTRLDKWSDQ
>XP030491963.1
MGSHHQNEEELENHDDTDTTPLLISSIEQADQPPDHITPSAHSSDSRLEKILSDTELPLT

NRLWQASCIEFKLLFRLAAPAIFVYLINNSMSLSTRIYAGHLGNLELAASSLGNSGIQLF
AYGLMLGMGSAVETLCGQAYGANRYDMLGIYLQRATVVLTITGLPLTIVYVLSKPLLLWL
GEPLELASAAALFVYGLIPQIFAYAVNFPIQKFLQSQSIVTPSAYISAITLIFHLVACWA
VAFELGFGLIGISLVLSLSWWIIVGAQFGYILVSDKCKMTWNGFSVEAFSGLWEFFKLSG
ASAVMLCLETWYFQVLVLITGLLDNPQLALDSIAVCMAINGLVFMVSVGFNAAASVRVSN
ELGAGNAKSAAFAVKVVNLISFLIAVAEAILVLSLRHVISYAFTGGDTVAEAVSDLCPFL
AVTLVLNGVQPVLSGVAVGCGWQAFVAYVNVGCYYVVGVPLGCLLGFKFDLGVKGIWSGM
IGGTMMQTFILLWVTFRTDWNKEVDKAKSRLEKWGDKKQPLLNK
>XP030484940.1
MDSSANDVDNRRPLLQQPLLNHDQYDSSSPQIILSSFGKGHHESSGQLETILSNTNVPFA
QRFWPALKIELRLLFFLAAPAVIVYMINYLMSMSTQIISGHLGNLELAASSLGNNGVQIF
AYGLMLGMGSAVETLCGQAFGAQKYGMLGVYLQRSTILLTLTGILLTFVYIFSKRFLVFL
GESERIASAAALFIYGLIPQIFAYAVNFPIQKFLQAQSIVAPSAYISAATLVVHVVLSWV
AVYKIGMGLLGASLVLSFSWWIIVVAQFIYIVKSERCKETWGGFSWEAFSGLPKFFKLSA
ASAVMLCLETWYFQILVLLAGLLENPELALDSLSICTTISGWVFMISVGFNAAASVRVSN
ELGGANPKSAAFSVIVVTVISFVISVIAAIVVLALRDVISYAFTDGETVAAAVSDLCPLL
SLTLLLNGVQPVLSGVAVGCGWQTLVAYINVGSYYVVGIPLGVLLGFYFNLGAKGIWLGM
IGGTVLQTLILLWITFRTDWTKEVEESLKRLNKWDDQTNKKQILNT
>XP030508827.1
MGSNNNDNSKNGGELEERLLENEIEINEGDLKWRVWREMKLIWKIAFPSMLARTTSFGVL
VVTQSFLGHVAELDLAAFALVQTLFLRFINGIILGMSSATETLCGQAYGAKQYHMMGIYL
QRSWIVNIITATITFPLFIFSTQILRALGEEEDIAVATGQISIWCIPYLYYFVFSFTIQM
YLQAQLKNMVVGWLSASSFVLHVLLSWFFVYKLNWGVAGALGGLNIASWASVIGQFIYIF
GGWCPNSWKGFTTAAFSDMWPVVKLSVSSGLMLCLELWYNSILVLLAGYMKDATTAISAF
SICLNISAWEFMICLGFLGAACVRVSNELGRGNAKAAKFAIKVIMGTSIVIGVISWILCL
IFGRQISYIFTDDEKVADAVADLSVLLAFSILLNSIYPVLSGVAVGAGLQGTVAIVNIFC
YYLIGIPLGVVLAYVINLEVKGLWIGMLCGVLAQTITLSIITWKTDWDLQVRKASERLNR
WFLKPDDELS
>XP030497193.1
MIEEEEVPLLKYQQEPRSEYLVTRTWLESKKIWQIAGPSIFSRLAMFSVTIVTQSFAGHF
SDVDLAAISIVTTVIVGITFGFLLGMASALETLCGQAYGGKKYHMMGIYMQRSWIVLFIS
SVLLVPMFIFPAPIMKLIGQTEAVAEKTGLVAKWLIPFHLSFPFQFSLQRFLQSQLKMAP
IAWISGGVLGLHVILSWVFVYKMRVGLVGAALTIDFAWWLSVLGMLGYTVYGGCPETWTG
LSSQAFVGLWDFIKLSLASGIMLSLENFYYRMLVIVSGYFQNTHIAVDALSICVTIYGWE
SMIPLGFLAATGVRVANELGAGNAKGAKFATMISVFTSICVGLGFCLIVVLLHEKLAMIF
TSTLPVIKMVNELAIMLVFSILFNCIQPVLSGVAVGSGWQSKAAFVNMGSYYLVGVPLGV
FFGWYLSLGIMGIWAGMLGGTIVQTVLLIIITMRCEWEKEVQKAWMNIIKEAASNCNN
>XP030482625.1
MGDDEQQYQPLLLGLESHSRIPDLSSVAVEEFLEQRPVACRWWPRLVCWESRLLWLLSGA
SIIVSIFNYMLSFVTLMFTGHLGALELAGASIASVGIQGLAYGIMLGMASAVQTVCGQAY
GAKQYSAMGIICQRALILHLGAAFLLTFLYWFSGPILIGMGQSKSIAEQGQVFARGLIPQ
IYAFALSCPMQRFLQAQNIVNPLAFMSAAVFILHVVLTWVAVYVLNYGLLGAALTLSLSW
WLLAIVNGLYILFSPSCKETWTGFSSKAFKGIWPYFKLTVSSAVMLCLEIWYSQGLVLIS
GLLTNPTVSLDSISICMNYLNWDMQFMLGLSAAASVRVSNELGAGHPRVAKFSVFVVSGT
SILISIVFSAIVLIFKVGLSKLFTTDSEVIEAVSELTPLLAISVFLNGIQPILSGVAIGS
GWQGVVAYVNLTTYYIIGLPIGCVLGFKTSLGVAGIWWGMIIGVLLQTLTLIILTARTNW
HNEVAKAADRLKASANEENSNLIISA
>XP030484051.1
MAAEDVDQSRLSLLRSSDPNYNDDDTKISDLSSEQVEEVLGKKPLPLRWWFRLVAWETRL
LWLLSGATIAVSEFNYMLTFVTLIFCGHLNALELAGASISSVGIQGLAYGIMLGMASAVQ
TVCGQAYGSKQYKAMGIICQRAIVLHLGAAVLLTFLYWFSGPILIAIGQSQSIAEQGQVF
ARGLIPQIYAFALTCPMQRFLQAQTIVNPLAYMSVGVFVFHVFLSWIVVDVFGLGLLGAA
LSLSFSWWLLVISNGLYILLSPTCNQTWTGFSWKAFTGIWPYFKLTLSSAVMLCLEIWYN
QGLVLISGLLSNPTLSLDSITICMNYLNWDICFMLGLGTSASVRVSNELGAANPKVAKFS
VLVVNGNTIIISLIFSAIVLIFRVALSELFTSDTSIIEAVSNLTPLLAISIFLNGIQPIL
SGVAIGSGWQAVVAYVNLTAYYVIGLPIGCVLGFKTSLGVAGIWWGMIAGVFLQTVTLII
LTAKTNWDSEVIKAADRVRRSTNETNLNFVVGV

>XP030497206.1
MASALETLCGQAYGGKKYHMMGIYMQRSWIVLFISSVLLVPMFIFPAPIMKLIGQTEAVA
EKTGLVAKWLIPFHLSFPFQFSLQRFLQSQLKMAPIAWISGGVLGLHVILSWVFVYKMRV
GLVGAALTIDFAWWLSVLGMLGYTVYGGCPETWTGLSSQAFVGLWDFIKLSLASGIMLSL
ENFYYRMLVIVSGYFQNTHIAVDALSICVTIYGWESMIPLGFLAATGVRVANELGAGNAK
GAKFATMISVFTSICVGLGFCLIVVLLHEKLAMIFTSTLPVIKMVNELAIMLVFSILFNC
IQPVLSGVAVGSGWQSKAAFVNMGSYYLVGVPLGVFFGWYLSLGIMGIWAGMLGGTIVQT
VLLIIITMRCEWEKEVQKAWMNIIKEAASNCNN
>XP030484052.1
MAAEDVDQSRLSLLRSSDPNYNDDDTKISDLSSEQVEEVLGKKPLPLRWWFRLVAWETRL
LWLLSGATIAVSEFNYMLTFVTLIFCGHLNALELAGASISSVGIQGLAYGIMLGMASAVQ
TVCGQAYGSKQYKAMGIICQRAIVLHLGAAVLLTFLYWFSGPILIAIGQSQSIAEQGQVF
ARGLIPQIYAFALTCPMQRFLQAQTIVNPLAYIPTCNQTWTGFSWKAFTGIWPYFKLTLS
SAVMLCLEIWYNQGLVLISGLLSNPTLSLDSITICMNYLNWDICFMLGLGTSASVRVSNE
LGAANPKVAKFSVLVVNGNTIIISLIFSAIVLIFRVALSELFTSDTSIIEAVSNLTPLLA
ISIFLNGIQPILSGVAIGSGWQAVVAYVNLTAYYVIGLPIGCVLGFKTSLGVAGIWWGMI
AGVFLQTVTLIILTAKTNWDSEVIKAADRVRRSTNETNLNFVVGV
>XP030492740.1
MEKMYWREEQKQSLNSPLIQVCGETEVENGVRRKEIVEEVKKQLWLAGPLISVSLLQFCL
QMISVMFVGHLGELTLSGASMATSFASVTGFSLLMGMASALDTLSGQSYGAKQYHMMGIL
MQRAILVLVVVSIPLAVIWANTEPILVTLGQDPAIAAEAGKYAKFMIPSIFAYGLLQCLV
RFLQTQNIVFPMMVSSGMTTLIHILVCWVLVFKSGLGSRGAAMANSISYWINVLLLALYV
MYSPSCAKTWTGFSKEALRNVATFVRLAIPSAVMVCLEMWSFEMMVLLSGLLPNPTLETS
VLSVSLNTASTVWMIPFGLSGAVSTRVSNELGAGRPDAARLAVCVVLVMAITEGLLIGLV
FILVRNVWGYAYSNQVEVVKYVAAMMPILAISNFLDGLQCVLSGTCRGCGWQKIGAYVNL
GSYYLVGIPSAVLMAFVLHVGGKGLWLGIICALIVQVISLFTFTIKTDWEQEAKKATDRV
YDSIVPVQLVS
>XP030492527.1
MSSNKVPLLEDLKTQDNNNNDDYEELSLPCRVKIESKKLWHIVGPAIFSRIASYSMLVIT
QAFAGHLGDLELAAISIANNVIVGFDFGLLLGMASALETLCGQAYGAKKYYMLGVYMQRS
WIVLSVCCVLLLPIYLFATPVLKVLGQPHDVAELSGVAAMCMIPLHFSFAFQFPLQRFLQ
SQLKTGVIAWVSLAALIMHIFISWLFVYQFKLGVIGTALTINFSWWTLVFGLLGYTLFGG
CPTTWAGFSIEAFSGLWEFTKLSTSSGVMLCLENWYYRILILMTGNLKNAEIALDALSIC
MTINGWEMMIPLAFFAGTG
>XP030499345.1
MNTINQTMEEERSLLLKPHISSENHEIIHWKVFFEEVKKLGYLGGPLVIVVLSQYLLQVI
SIMMVGHLGELALSSSAIANSLSVVTGLSVLMGMASALETICGQAYGAKQYKKLGLQTYT
SIFCLFLVCIPISLVWIFMGKILVFIGQDPQISHEAGKFIIWLIPALFAYAILQPLVRYF
QTQSMITPMVITSFAILCFHIPLCWVLVFKFGMGNVGGALAISISYWLNAIILGFYIKFS
PLCEKTRTPISMEMFHGIGEFFRLAIPSAIMICLEWWSFELLILMSGILPNPQLETSVLS
VCLQTISTLYAIPYGVGAAASTRVSNELGAGNPQGARIATFAAMFLAIAETSIVTTTLFM
SRGIFGYTFSNEKEVVNYVTEMAPLVCLSVVLDSMQGVLSGIARGCGWQHIGAFINLGAF
YLCGIPIAAILGFWVNMRGKGLWIGIQSGALVQTTLLALVTGCTNWEKQASMAKERLFGD
TSKNGLEH
>XP030499346.1
MNTINQTMEEERSLLLKPHISSENHEIIHWKVFFEEVKKLGYLGGPLVIVVLSQYLLQVI
SIMMVGHLGELALSSSAIANSLSVVTGLSVLMGMASALETICGQAYGAKQYKKLGLQTYT
SIFCLFLVCIPISLVWIFMGKILVFIGQDPQISHEAGKFIIWLIPALFAYAILQPLVRYF
QTQSMITPMVITSFAILCFHIPLCWVLVFKFGMGNVGGALAISISYWLNAIILGFYIKFS
PLCEKTRTPISMEMFHGIGEFFRLAIPSAIMICLEWWSFELLILMSGILPNPQLETSVLS
VCLQTISTLYAIPYGVGAAASTRVSNELGAGNPQGARIATFAAMFLAIAETSIVTTTLFM
SRGIFGYTFSNEKEVVNYVTEMAPLVCLSVVLDSMQGVLSGIARGCGWQHIGAFINLGAF
YLCGIPIAAILGFWVNMRGKGLWIGIQSGALVQTTLLALVTGCTNWEKQASMAKERLFGD
TSKNGLED
>XP030499347.1
MEESLLLLEHSNHQEEDISKRNEIIHWKVFFEEVKKLGYLGGPLVIVLLCQYFLQVISMM
MVGHLGQLPLSSSAIAISLSGVTGFSLLLGMASALETICGQAYGAKQYKKLGLQTYTSIF

CLFLVCIPISLVWIFMGKILVFIGQDPQISHEAGKFIIWLIPALFAYATLQPLIRYFQTQ
SMITPMVITSFAILCFHIPLSWVLVFKFGLGNLGGALGIGISYWLNAVILGFYIKFSPLC
EKTRAPISMEIFRGIKQFFRLAIPSAIMICLEWWSFELLILMSGILPNPQLETSVLSICL
QTIATLYAIPYGVGAAVSTRVSNELGAGNPQGARIATFAAIFLAIAEMSIVTTTLFMSRS
VFGYTFSNEKEVVSYVTDMAPLICLSIVLDSIQGIMSGIARGCGWQHIGAFINLGAFYLC
GIPIAAILGFWANLRGKGLWIGIQSGAFLQTILLCLVTGFTNWEKQASNAKERLYGDGTK
NVLVH
>XP030508962.1
MIYRQSSKNSIVLVMEENLKEEKNKWEISWRSLVREMKREGIIAGPMVGVAVLQYLLQVS
SLIMVGHLGHLSIASVAIAISLTNVTGFSLLSGMAGGLETLCGQAFGAEQYHKLGTYTYA
AIISLSLICLPISVLWIFMDKFLFLVGQDPLISIEACHFSMCLIPGLFASAILKPLTRYC
QTQSSILPMLLITLVVLVFHILTCWILIYKFEIGKNGAAISISLSTWLNVMLLGFYVTYS
SVFEKTRISFSKEAFQGVGEFYRYGVPSAVMVCLKWWSCELLILLSGLLPNPNLETSVLS
ICLTISTLHFTISYGVGATTSTRVSNELGAGKPEAARLAVWAAMFIAATEAVTVSISLFL
CRSFVGYAFTNEKQVVSYIAIMAPLICLSVFMDSLQAVLSGVARGSGWQRMGAYINLGAF
YVAGLPMGIVLGFALHLKGKGLWIGIVIGSVVQSTLLFLVTIFSNWKKQAIKARERAIST
LAEH
>XP030499344.1
MTMEESLLLKNNVHEEQRDINGDIEIENKIVVTLWNWKVFFEEVKRLGFLGGPLVIVVLS
QFLLQVISIMMVGHLGELALSSSAIAISLSGVTGFSVLMGMASGLETICGQAYGAKQYKK
LGLQTYTAIFCLFLVCIPISLIWIFMGKILVFIGQDPQISHEAGKFIIWLIPALFAYAIL
QPLIRYFQTQSMITPMLITSFAILCFHIPLSWVLVFKFGLGNLGGALGISISYWLNAIIF
GFYIKFSPSCEKTRTSISIEIFHGIREFFRLAIPSAVMSCLEWWSFELLILMSGILPNPQ
LETSVLSICLQTISTLYAIPFGVGAAASTRVANELGAGNPQGARIATFAAMFIAIVETSI
VTTTLFMCRDVFGYTFSNEKEVVSYVTEMAPLLCLSVVLDSMQGNLSGIARGCGWQHIGA
FINLGAFYLCGIPIGVILGFWANLRGKGLWIGIQIGSFVQIILLSFVTGCTNWEKQASMA
KERLFGDTSKNGLED
>XP030509465.1
MEEALLLVPEDKGCCGLGKEIKKLSYIAAPMVVVSVSQNLLQVVSVMMAGHLGELSLSGV
AIASSFTNVTGFSLLFGMAGGLETLCGQAYGAEQYKKLGSYTYCSIISLIFVCFPVSLLW
MFVEKILIMMGQDPLVSSEAGKYSIWLLPALLAYAILQSLIRYFLTQGLILPMLLSSCAT
LCFHVPLCWALVYKFELGTTGAALSIGFSYWLTVIFLVIYIQYSSACDKTRVSFSLDVFR
SIPEFFRFGFPSAVMSCLEWWSFELLILFSGLLPNSKLETSVLSICLTTMSLHYFIPYGV
GAAASTRVSNELGAGNPRGAKAAVLAATMIAAVEAISVSTILFSCRSVLGYAYSNEKEIV
DYVTRITPLLSFSVIMDSVQAVFSGVARGTGSQRLAAFVNLGAYYLVGLPLALVLCFMLQ
LRGMGLWAGILAGTTTQALFYALITSFTNWQKKAVEARERIFEGTNVGESVFA
>XP030498726.1
MDEKGENMEKGLLEKEKEKESIFSKVFDEVKLLGYIGGPMVIVNMSQYVLQIISIMMVGH
HGKLALSSTAIAISFSAVSGFSLIFGMSSALETLCGQAYGAKQYRKLGILTNTGILTLTL
VCIPLSLMWSYMENVLVFMGQDPIISREAGKFSIWLIPALFAYATLQPLVRYFMTQSITY
PLLLSSCASICCHLPICWALVFKSRLGHFGAALSIGFSYWLNVSLLLLFMKFSPACEKTR
ASIFSNLFQGVAEFLRFGIPSSLMICLEWWAFELLTLLSGFLSNPQLETSVLSVCLSTVI
TLYTIPDGFGAAISTRVSNELGAGKPRAAQFAVRVILCLVAIEAVIVSSSLFLGRNTFGY
VFSNEKEVVHYVTSMAPLVCATLILNSLQGVLSGIIRGCGWQDLGAFVNLGSYYFFGIPA
AAALGFWLKMRGKGLWIGICSGCFLQIILLSIITFFINWEDKANLARKRVFKKRSIEDNG
LSEHNNSSLY
>XP030498725.1
MEEKREISMEKGLLEKEEKEISIFSKIFDEVKLLGYIGGPMVIVNISQYFLQIVSVMMVG
HHGKLALSSTAIAISFSAVSGFSLILGMSSALETLCGQAYGAKQYRKLGILTNTGILTLI
LVSIPLSLMWFYMEKILVFMGQDPIISREAGKFSIWLIPALFAYATLQPLVRYFMTQSIT
YPLLLSSFASICCHLPICWALVFKSRLGHFGAALSIGFSYWLNVSLLLLFMKFSPTCEKT
RASIFSNLFQGVGEFLRFAIPSSLMICLEWWAFELLTLLSGFLSNPQLETSVLSVCLSTI
ITLYTIPDGFGAATSTRVSNELGAGKPKAARFVVRVVLCLVVIEAVIVSSSLFLGRKTFG
YVFSNDKEVVDYVTSMAPLVCAAVMINSLQAILSGIIRGCGWQDLGAFINLGSYYLFGIP
TAATLGFWLKMRGKGLWIGICCGCFLQMILLSIITIFINWEEKAKLARERVFKKRSLEDN
VLSEHDNSNLY
>XP030508611.1

MEADHEDEVPLLVKRGKSSELLLGGVREGRFVGEMKKMIAIGMPMVVVTVAQFLPQVVST
MMAGHLGELSLSGAAIATSFADVTGFCLLLGMAGALETLCGQTYGAEEYHKLGEYTWCAI
ISLTFICLPISLLWIYMDRVLIWVGQDPLISQAAAQYSTCLIPALFGYAVLQSLVRFFQS
QRLILPMLISSCSVLCLHIPLCWILVFKLHLGISGAAFSIGGSYWLNVVFLGLYMKYSSA
CEKTRIKFSMEAFRHIREFMRLAIPSALMLCLEYASFELLVFLSGLFPNAKLEASVLSIC
ITTTTSIFYIPYGIGAATSTQVSNRLGAGNAEGAKPAIKSALVLGVAEAFLGSTILFCCR
NVLGYAYSNEIEVVNYVKEMVPLLSFSIGVDSLVAVLSGVARGSGWQDIGAYVNLGAYYV
VGIPLAALLGFVFKLRGKGLWIGIMTGTTLQALFFLVITVSTNWQNQAEKARERIFKENE
TLINHSVPEYYVEEQKTLIC
>XP030496616.1
MGESLMRKQEEEQNLEKGLLENEKREEKVEFFRDFIEELKLLGYIAGPMVAVNLSLYFLQ
IISLMMVGHLGKLYLSSTAIAISIGAVSGFSFLFGMSGALETLSGQAYGAKQYRKLGTQT
NTAILCLILVCIPLSLVWFYMENILILIGQDPHIXREAGQFLLWLIPALFAYATLQPLIR
YFQSQSLIKPLLVSSCVSICCHVPLCWTLVFKCGLQHCGAAISIGISYWLNVILLVLYMT
FSSSCKSTRVPISPELFQGFGEFFRFAIPSAAMSCLEWWSFEIITLLSGLLPNPQLETSV
LSVCMSTIATLYTIPEGLGSAGSTRVSNELGAGKPRAARLAVGAVMFLSVSESIIVSSAL
FAGRKAFGYFFSNDKEVVDYAMNMAPLVCLSVIFDSLHATLSGIARGCGWQDLGAFVNLG
AYYLVGIPVAAALGFWFDLRGKGLWIGIVVGSFLQAFLLSLIAVCTNWDEKAKMARKRIF
EDSTTSLS
>XP030499266.1
MGESLIWKQEDEDNLEKGLLENEKREEKVEFFRDFIKELKLLGYIAGPMVAVNLSLYFLQ
IISLMMVGHLGKLYLSSTAVAISIGAVSGFSLLFGMSGALETLSGQAYGAKQYRKLGTQT
NTAILCLILVCIPLSLIWFYMENILIVIGQNPNISRQAGQFLLWLIPALFAYATLQPLIR
YFQSQSLIKPLLVSSCVSISCHVPLCWTLVFKCGLQHCGAAISIGISYWLNVILLLLYMT
FSSSCESTRVPISSELFQGCGEFFRLAIPSAAMSCLEWWSFEIITLLSGLLSNPQLETSV
LSVCMSTISTLYTIPEGLGSAGSTRVSNELGAGKPRAARLAVGAVMFLSVSESIIVSSAL
FAGRKAFGYFFTNDKEVVDYAMNMAPLVCLSVIFDSLHATLSGIARGCGWQDLGAYVNLG
AYYLVGIPVAAALGFRFDLRGKGLWIGIIIGSFLQAFLLSLIAICTNWNEKAKMARKRMF
EEIFEESTTSLV
>XP030486933.1
MAGNSDAREPLIEVEEIEVSRRKWWDRLLDAQEAKQQVLFSLPMIITNGFYYLIPLVSVM
FAGHLGQLNLAGATLANSWATVTGYAFMIGLSGALETLCGQGFGAKSFKLLGIHLQTACI
TSTLFSIIISFLWFFTEPLLIFLHQEHQIAETAGLYTKFLIPSIFAFGYIQNILRFLQTQ
SVVRPLVLFSSIPLVFHIGITYCLVHLTPLGFKGAPLATSVTLWLTFLMLAAYVMFSKEF
KLTWQGFSLESFSYILTNLKLALPSATMVCLEYWAFEIMVFLAGLMPNSEVTTSLIAMCV
NTESIAYSILYGLSAAASTRVSNELGADRPDKAKNAMIVTLKLCGLLGFVLVLALGFGHN
IWAGLFSDSSDIIKSFASMTPLLAISIIVDSFQGVLSGVARGCGWQHMVVYVNLATFYFI
GMTVAAILGFKFEFYAKGLWIGLICGLSCQAVTLMVITMRAQWTKLQLSEDKSEKEYSIS
A
>XP030488163.1
MEGISSSISNRNIVAALPRDLSGTAGSSSDGGEERGNVIIVNITSTSSGEKWWKKVLEVE
EAKQQVLFSLPLILTNGFYYLIPLISVMFASHLGPLQLAGATLANSWATVTGYAFLIGLS
GALETLCGQGFGANLYKMMGIHLQASCITSFLFSILISFVWFNTEPILVFLGQDLEIAET
AGIYTKFLIPSIFAFGYIQNILRFLQTQSVVRPAVLLSAIPLVFHIGITYCLVHLTPLGF
KGAPIATSITLWISFFMLAAYVLLARQFNLTWQGFSTESFSYILTNLKLSLPSAAMVCLE
YWAFEILVFLAGLMPNSKSTTSLIAICVNTETIAYNVTYGLSASASTRVSNELGGGHPEQ
AKSAMVVSLKLSVLLATIVVMALGFGHNFWAGLFTDNSAIIKSFASMTPLLAISIMLDSL
QGVLSGVARGCGWQRMVVYVNLITFYLVGVTVGSLLAFKMKLYVKGLWIGLICGLACQAA
TLVLITMCTNWHMIAAVV
>XP030509466.1
MEEALLLVPEDKGCCGLGKEIKKLSYIAAPMVVVSVSQNLLQVVSVMMAGHLGELSLSGV
AIASSFTNVTGFSLLFGMAGGLETLCGQAYGAEQYKKLGSYTYCSIISLIFVCFPVSLLW
MFVEKILIMMGQDPLVSSEAGKYSIWLLPALLAYAILQSLIRYFLTQGLILPMLLSSCAT
LCFHVPLCWALYSSACDKTRVSFSLDVFRSIPEFFRFGFPSAVMSCLEWWSFELLILFSG
LLPNSKLETSVLSICLTTMSLHYFIPYGVGAAASTRVSNELGAGNPRGAKAAVLAATMIA
AVEAISVSTILFSCRSVLGYAYSNEKEIVDYVTRITPLLSFSVIMDSVQAVFSGVARGTG
SQRLAAFVNLGAYYLVGLPLALVLCFMLQLRGMGLWAGILAGTTTQALFYALITSFTNWQ

KKAVEARERIFEGTNVGESVFA
>XP030488086.1
MENSDVREPLIEVEGNEVIRGKWWNRVLDADEAKQQVLFSLPMIITNGFYYLIPLISVMF
AGHLGQLHLAGATLANSWATVSGYAFMVGLSGALETLCGQGFGAKSFKLLGIHLQTACIT
STIFSIIISFIWFFTEQLLLFLRQDPQIAETAGLYTKFLIPSIFAYGYIQNILRFLQTQS
VVRPLILFSSIPLVFHIGITYCLVHLTPLGFKGAPLATSITLWLTFFLLVAYVMFSKEFK
LTWHGFSLESFCYIYTNLKLAIPSAAMVCLEYWAFEILVFLAGLMPNSEVTTSLIAICVN
TEAITFNFTYGLSAAASTRVSNELGAGRPDRAKSAMIVTLKLCGVLALILVLALGFGHNI
WAGLFSDSSEIIKSFASLTPLLAISIVIDSLQGVLSGVARGCGWQQTAVYYVNLGTFYFIG
MTVAAILAFKFELYVKGLWIGMTCGMLCQAFALMLITMCTQWTKLQLPEEKPKKGDSILA
>XP030487456.1
MAGNSEAREALIEVEEGEVINRGKWWDRVLDAEEAKQQVLFSLPMILTNGFYYLIPLISV
MFAGHLGHLHLAGATLANSWATVSGYAFMIGLSGALETLCGQGFGAKSFKLLGIHLQTAC
ITTTLFSIIISFLWFFTEQLLIFLRQDPQIAEAAGLYTKFLIPSIFAYGYIQNMLRFLQT
QSVVRPAVLLSAIPLVLHIGITYCLVHLTPLGFVGAPLATSITLWLSFFMLVAYVMFSKE
FNLTWQGFSLESFSYILTNLKLALPSATMVCLEYWAFEILVFLAGLMPNSEVTTSLIAIC
VNTEAIAYNFTYGLSAAASTRVSNELGADRPDKAKSAMIVSLKLCGVFALILILALGFGH
NIWAGTYSDSSDIIKSFASMTPLLAISMTVDSFQGVLSGVARGCGWQHIVVYYVNLATYYI
VGMTVAAILAFKFELYVKVLAM
>XP030502200.1
MILTGLLLYSRSMISMLFLGRLGELALAGGSLAVGFANITGYSILSGLAMGMEPICGQAF
GAKKNTLLGLCLQRTILLLMLTSIPISLLWLNMKKILILCGQDEAIAMEAQSYLLYSLPD
LLAQSLLHPLRIYLRTQSITLPLTYCATFSIFLHIPINYFFVSHLNWGIKGVALSGVLTN
FNLVASLILYILVSGAHKKTWGGISTDCFKDWKTLLNLAIPSCISVCLEWWWYEIMILLC
GLLINPRATVASMGILIQTTALIYIFPSSLSFSVSTRVGNELGANKPKKARFAAIVGLSC
SFALGLVALVFAVMVRKVWATMFTQDKDIISLTSLVLPIIGLCELGNCPQTTGCGVLRGT
ARPKVGANINLGCFYMVGMPVALVLSFFAGFDFKGLWLGLLAAQGCCALTMLVVLGQTDW
NSEAQRAKLLTGAQIIDHGDDDDDHGDDDSELVDEEKPNEKSVLVKGDGLYTTLCDCKNN
SPV
>XP030495219.1
MCHPKPSSPLSSFLCFKTALLMNHRKPVDPHYPEDQHELHRWPTPSEVLEEVKAIGKISG
PTALTGLLLYSRAMISMLFLGYLGELQLAGGSLAIGFANITGYSVISGLAMGMEPICGQA
YGAKQWKLLGLTLQRTVLLLLSTSIPISFMWLNMKNILLLCGQDHEISSMAHTFILFSVP
DLCFLSLLHPLRIYLRTQSITLPLTYCTAISVLLHVPLNFILVVKLQMGISGVAIAMVWT
NLHQFLLLGGFVYFSGVYKDSWVSPSIDCLRGWSSLLSMAVPTCISVCLEWWWYEFMILL
CGLLGNPKATVASMGIMIQTTSLVYVFPSSLSQGVSTRVSHELGANRPGKARISMIVSMA
LAVFAGLTAMIFTTSMRYQWGRLFTDDAEILKLTAIALPIVGLCELGNCPQTTGCGVLRG
SARPTVGANINLGSFYLVGMPVAVFIGFFLKLGFPGLWLGLLAAQGCCALLMIYVLCTTD
WRLQVERAINLTKDEQYCDDDTRSTNYNNINNLNNNNNKNENIEGPPLETISSSTSAPDD
SMIIRERNHNKRGTNDLEEIIVVCINDNDDDGDDEDDDLPFNSTESPETYPLLISQATRT
>XP030500350.1
MCHHSSSPSSCLCDYDSNKPKVLSVTVQDSNERTPLINSKTTDLLESQTQNSQRSHLSEA
VSEAKCIANIALPMVLTGLLLYSRSLISMLFLGRLGELSLAGGSLAIGFANITGYSVLSG
LAMGMEPICGQAFGAKRFKLLGLTLQRTVILLLLTSLPIAILWLNMRRILLLCGQQDDIA
TEAQSYIFFSLPDLIAQSILHPLRVYLRTQSITLPLTFCATLSIILHIPINYFLVSVLNL
GIKGVALSGVWTNFNLVGSLIVYVVISGVYKKTWVEISSDCLRAWKSLLSLAIPSCISVC
LEWWWYEFMILLCGLLLNPQATVASMGILIQTTALMYIFPSSLSFGVSTRVGNELGANNP
QRAKLAAIVGLLYSSVLGLSALLFAVMVRNIWAKMFTQDVEIIALTSMVLPIIGLCELGN
CPQTTGCGVLRGTARPSFGANINLGCFYLVGMPVAIWLSFYVGFDFTGLWLGLLAAQSSC
VVTMLVVLARTNWEIQAQRAKELTGSHVTLEEVDHDHNHDNYDDDEVKDSFCSLHFAESD
DHSSSNNNNNSQV
>XP030488326.1
MEQKHQDFSFTKFPTLSQIIQELKQLWSLALPITGMNLLVFYRAAVSVFFLGQLGRLELA
GGTLSLGFTNITGYSVLMGLATGLEPVCSQAYGTKNQPLLAISFQRMVLILTLASIPITL
LWLNLEKIMISMGQNREITSMAATYCFYSLPDLFTNSLLQPLRVFLRSQEAVKPMMYCTM
AAVFLHAPLNVVMVKKMGLGVGGVAAASVMTNLNMVLLMGGWYLGWGGGWEMVRLFGGFK
EVCMGIGGLLRLAVPSCLGICLEWWWYEIVTVLAGYLDNPTLAVAATGILIQTTSMMYTL

PLALSGCVSTRVGNELGAGNPYKAKLAAMVALGCAFLIGFINVTWTVILRERWAGLFTKD
DLVMALVASVMPIMGLCELGNCPQTTGCGILRGTARPVVGARVNLGSFYFVGTPVAVGLA
FWLKIGFPGLWFGLMSAQLACVVSILYVVLLHTDWEAEALKAQKLAGVEMSTSACIEEGE
TKYEEDEESKGLLITNGNGNHNKDDVFWTE
>XP030487307.1
MCNPTTNTCTSSTTTKETEHNIKEPQTHLFLDLLSLSNTNIKDQEKTTSAKPDDDHYQQP
NSFTSDIISEAKSLFNLGFPIALTALILYSRSIISMLFLGHLGDFQLAAGALAIAFANIT
GYSVLSGLALGMEPLCSQAFGAQRPKLLSITLHRSIIFLLVSSLPISFLWLNMSRILFCL
NQDPSITTMAHTYLLFSLPDLFTNSFIHPIRIYLRSQGITVPLTLASLAGTLLHFPLNFL
LVTRLKLGVAGVAAASSAVNLFVLLCLVSYVWWDSGLLEPAWSAPSRECLSGWKPLLRLA
APSCVSVCLEWWWYEIMIVLCGFLADPKSTVASMGVLIQTTSLIYVFPSSLGFAVSTRVG
NELGANRPYRAKLSAVVAVLLAFVTGLSAMSFAYGMKDTWGRMFTSDAEILRLTSAALPI
LGLCELGNCPQTVGCGVLRGSARPCTAANVNVSAFYLVGMPVAIGLSFWVGIGFCGLWMG
LLAAQICCAGLMLYVVGTTDWNFEAKRAQMLTCASCEDNILPSIDCKDDEEQPLICIVVT
SSK
>XP030508430.1
MVPQVEPENNKVQKYPTMPEVVEELKKVSNIGFPILTMSLVNYLRNMAMVICMGRLGSLE
LAGGALAIGFTNITGYSLLSGLAMGMDPLCSQAIGSQNHALAFLTLQRTIFILLFASLPI
SLLWFNLQPLMLILNQNPDITKIATIYCKFAIPDLLANSLLHPLRIFLRSKSITWPLMWS
NLLAMFLHIPITIFLTFKLSLGVQGIAISNFITNFITLFLLLLYLYFNTNFNKESYYSNS
NFTTNLKTPFLPKSWPTFFTNSTWEGWGVLIRLSIQSCLAVCLEWWWYEFMTLLAGYLPN
PKVALATSAIVIQTTSLMYTIPSALSASVSTRVGNELGAERPEKAKLAASVAVSLALITS
LVGLVGTTFGRGAWGRVYTHDNEILELTMAVLPIIGVCELANCPQTTSCGILRGCARPGI
GAAINFYAFYLVGAPVAVVLGFVWKLGFLGLCYGLLAAQIACVVSILTVVYNTNWEKELE
KAKDLMMIKDITVDNVHHNHDYDNSHGHDHITVLLQ
>XP030480622.1
MCPNSSDESQTIINGSLDEPLLPKQNGINMRPLVVEGGGDGGGGGGGVGGAATRFLQGFL
QRFFPANSLLRTLPLNEVSEELKSLVKIAWPIIMTTLLIYSRSIVSMLFLGRLGKTELAG
GSLALGFGNITGNSILRGLSTGMDPICCQAYGAKRFSVLSHAFQKTFCLLLLVSIPISAL
WLNMEPICIWLGQDPATIRVAKIYMAFSVPELLAQAHLHPLRIFLRTQGLTFPLTVAATC
SAILHLPINYFLVFHLKLGVAGVALALAWNTVNLNLGLLIYILFSNKPLKPWTGLTIFST
FQGWGPLLKLALPSCAAVCLEWWWYEIMMFLCGILKNPQASLASMGILIQTTGLLYVFPI
SLSAGLTTRVGHALGSGQPVRAQWTAILGLAFAFGFGLLALVLMIMVRSWWGRLYTDEPQ
VLDLLSKALPILGLCELGNTPQTAACGVLNGMARPNLGARINLCAFYGVGLPVAVLVTFK
FRYGFLGLWIGLLSAQISCLGMMVYVLVQTDWKHQSKRAEEMVSGTDEENQKNPEK
>XP030495192.1
MVCKYEWGSSGAAVATSISYCVNAFCLALFIKFSSTCKETWNGFSKQALHNFLPYMKLAL
PSVLMECFKTWAFEILIIFSGFFPNPVLETSVQSICLNTFGLIWSIPAGLMVSASVRVGN
EIGAKNLEAARLAMVVVSVIVIIEGLVVGISIILLRKMWAGLYSNDENVVKIVSSLMPFL
AMSCFLNGFQTVLSGIFKGCGWQNKGVYLNLAAFYFVGIPCQVTFAFVLHMKSKGLWLGM
IIGFVVNVIFFLMIILRANWDKEVMKAENRVKECSTYGEKSSGQNNSS
>XP030498085.1
MHVISTHPLLSPLYGKEGYGIIGDHSKRIQDTLSKVLEEMKELYVIAIPMIMAGLLVYGK
SLISLFFMGMIGKDALTGGSLAICVANITGYSVVSGLAAGMEGISSQAYGANKWSLMGQT
LNRTILMILTLATFPISFSWFNSKPLFLYWFNNNQTLSTIATTYLKYSIPSLILQSFINP
LRIHLRTQKLTKPLMLSAALALASHAPINYYFINHLGFNNIGAVALSASLTDLNIFVSLA
FYLYFFGTMRWSWPEWSFRNCFLEWKPILSQAIPSCISVCLEWWWYELMIMLSGLLVNSG
DAVACMGILMQATMLVYQFPFALNQAASTRVGIELGANQPKKAKTAAFVALSCGFVTGIV
SLVFMVAVKDTWGKIFTKDRAILSLSAAVLPVVGLCEVGNCPQTTVCGVLRGSGRASMGA
CINVGSFYGVGFPVAMFVGFKMGKGLIGLWVGLVGAQLVCACVMVVVLVTTDWNVQAERA
KELTRSTSNDVDDGLISLVLEN

Previously described flavonoid transporter belonging to MATEs were included according to [35]: *Arabidopsis thaliana* FFT, (AT4G25640.2) and AtTT12 (AT3G59030.1); *Vitis vinifera* VvanthoMATE1, (NP_001290007.1) and VvanthoMATE3 (NP_001268037.1); *Medicago truncatula* MtMATE1, (XP_003592215.2) and MtMATE2 (; *Solanum lycopersicum* MTP77 (Solyc03g025220.2.1).

>SlMTP77
METPLLNGYSGSGERNDLIGADGDYRPAKSTKDWWAIFCVETLKLWRIGGPIAFNIICQY
GVNSLTNIFVGHLGNVELSAISIAQTVISTFSFGFMMGMGSALETLCGQAYGAGQVHMLG
VYMQRSIIILLATCVFLLPIYLFTTPLLVLLGQETAIADLSGRYTMLLIPQLFSLAINFP
TSKFLQAQSKVDVLAGIGFAAVLVHALFLWLFIYTLEWGTNGAAIAFDLTNWLTAMAQLA
YVVGWCKDGWKGLSWSAFNEIWAFVRLSIASAVMLCLEIWYMMSIILLVGHLNNAVIAVG
SISICMNINGWESMLFIGINAAISIRVSNELGQGHPRATKYSVYITVFQSLLIGILCMVI
VLVARDHLAIIFSNSKEMQEAVADLAYLLGITMVLNSVQPVISGVAVGGGWQALVAYINL
GCYYVFGLPLGYLLGYVAKLGTKGLWLGMIAGAALQTLLLLIILYKTNWNKEVNDTTERM
RKWGGQDFETQKSADGQLTIENGGVA
>AtTT12
MSSTETYEPLLTRLHSDSQITERSSPEIEEFLRRRGSTVTPRWWLKLAVWESKLLWTLSG
ASIVVSVLNYMLSFVTVMFTGHLGSLQLAGASIATVGIQGLAYGIMLGMASAVQTVCGQA
YGARQYSSMGIICQRAMVLHLAAAVFLTFLYWYSGPILKTMGQSVAIAHEGQIFARGMIP
QIYAFALACPMQRFLQAQNIVNPLAYMSLGVFLLHTLLTWLVTNVLDFGLLGAALILSFS
WWLLVAVNGMYILMSPNCKETWTGFSTRAFRGIWPYFKLTVASAVMLCLEIWYNQGLVII
SGLLSNPTISLDAISICMYYLNWDMQFMLGLSAAISVRVSNELGAGNPRVAMLSVVVVNI
TTVLISSVLCVIVLVFRVGLSKAFTSDAEVIAAVSDLFPLLAVSIFLNGIQPILSGVAIG
SGWQAVVAYVNLVTYYVIGLPIGCVLGFKTSLGVAGIWWGMIAGVILQTLTLIVLTLKTN
WTSEVENAAQRVKTSATENQEMANAGV
>VvanthoMATE1
METPLLKSGAERGYGGEGGDYPPLTTWREVRSMLWRETVKVWRVAGPLAFQILCQFGTNL
VTTVFVGHIGNLELSAVSISVSVIGTFSFGFMLGMGSALETLCGQAYGAGQVQLLGVYLQ
RSWIILLVSCIILLPIYIFATPILKALGQEDEIADLAGQFTLETIPQLFSLAIIFPTQKF
LQAQSKVNVQATICFVALILDIGMLAVFIFVFGWGTTGAAIAYDISSWVTAVAQVVYAIS
WCKEGWTGLTWSAFREIWAFVRLSLASAVMLCLEIWYFMIIILLTGHLQNAVIAVGSLSI
CMTFGGLEVVMFMGMNAAVSIRVSNELGYGHPRAAKYSVFVAVSQSLLIGIFCMVVVLLA
RDYIAIIFTTNKEMQEAVSHLAYLLGVTMLLNSLQPVFSGVAVGGGWQAMVAYINLGCYY
IIGIPLGYLLGYKAKLGVQGLWGGMICGTALQTLVLLFIVYRTNWNREVEQTTERMQKWG
GQRIEADDV
>VvanthoMATE3
METPLLNSGAEEGYSGPDGDYQPLRSWREVRSMVWKETVKLWRVAGPLAFQILCQFGTNS
MTSVFVGHIGNLQLSAVSISLSVIGTFSFGFMLGMGSALETLCGQAYGAGQVHLLGVYLQ
RSWIILLVTCVILSPVYVFATPILKVLGQEDAIADLAGQFTIETIPQLFSLAIIFPTQKF
LQAQSKVNVQATIAFVALILHIGMLSVFIFVFGWGTTGAAIAYDISNWVIAVSQVVYAIG
WCKEGWTGLTWSAFREIWAFVRLSIASAVMLCLEIWYFTIIVLLTGHLQNAVIAVGSLSI
CMTFNGWEGMLFIGMNAALSIRVSNELGYGHPRAAKFSVYVAVSQSLLIGILCMVVVLLA
RDYIAFIFTSNKEMQEAVSNLAYLLGATMLLNSMQPVLSGVAVGSGWQALVAYINLGCYY
IIGVPLGCLLGYLAKFGVKGLWGGMICGTALQTLILLFIVYRTNWNKEVEQTTERMQKWG
GVQIETKKTSDDV
>MtMATE2
MDSHTPLLNTTAATSSSSELLELDGGDYLEVKGFKQARKVFAIETLRIWKIALPIVFNIL
CQYGVNSITNIFVGHLGDIQLSAISLINSVIGTFAFGFMLGMGSATETLCGQAFGAGQVH
MLGVYMQRSWIILFVTSIILLPIYIFAAPILKLLGQQEDMADLAGSFALLVIPQFLSLSF
NFPTQKFLQSQSKVNVIAWIGLVALIVHIGLLWLLIYVLDLGLTGAAIAFDVTSWGITLA
QLVYVVIWCKDCWNGLSWLALKDIWAFVRLSVASAVMLCLEIWYMMSLIVLAGHLDNAVI
AVDSISICMNFNGWEGMIFIGVNAAISVRVSNELGLRHPRAAKYSVYVTVFQSLFMGIFF
MGVILVTKDYFAIVFTNSKTLQVAVADLGNLLAVTMVLNSVQPVISGVAVGGGWQALVAY
INVGCYYLFGLPLGYILGNVAELGVKGLWGGMICGILLQTLLLSGILYKTNWNKEVDNTS
ARVQQWGGQTVEVDSNGVDKP
>AtFFT
MDPTAPLLTHGGEVEEDYAPARSWTDVKRVLSTESAKLWMIAAPVGFNIICQYGVSSVT-
NIFVGHIGEVELSAVSISLSVIGTFSFGFLLGMGSALETLCGQAYGAGQVNMLGVYMQRSWIILFVSCFFLLPIYIFATPVLRLLGQ
AEEIAVPAGQFTLLTIPQLFSLAFNFPTSKFLQAQSKVVAIAWIGFVALSLHVIMLWL-
FIIEFGWGTNGAALAFNITNWGTAIAQIVYVIGWCNEGWTGLSWLAFKEIWAFVRLSIASAVMLCLEIWYMMSIIVLTGRLDNA

VIAVDSLSICMNINGLEAMLFIGINAAIS-
VRVSNELGLGRPRAAKYSVYVTVFQSLLIGLVFMVAIIIARDHFAIIFTSSKVLQRAVSKLAYLLGITMVLNSVQPVVSGVAVGGG
WQGLVAYINLGCYYIFGLPFGYLLGYIANFGVMGLWSGMIAGTALQTLLLLI-
VLYKTNWNKEVEETMERMKKWGGSETTSKDILASGWPSGLRRQTQVLVFVRGRGFKPHF
